# Comparative genomics of Eastern-Indian *Ustilaginoidea virens* strain NRRI-FSM-1 at whole-genome level unravels genome evolution and genetic plasticity

**DOI:** 10.3389/ffunb.2026.1828327

**Published:** 2026-05-21

**Authors:** Basavantraya Navadagi Devanna, Pankaj Kumar Singh, Himanshu Dubey, Sanghamitra Samantaray, C. Parameswaran, Lambodar Behera, Manas Kumar Bag

**Affiliations:** 1 Indian Council of Agricultural Research (ICAR)-Central Rice Research Institute, Cuttack, India; 2 Department of Biotechnology, University Centre for Research and Development, Chandigarh University, Mohali, Punjab, India; 3 Central Silk Board (CSB)-Institute for Seri-Biotechnological Research, Bengaluru, India

**Keywords:** comparative genomics, Eastern India, genome evolution, rice false smut, whole genome sequence

## Abstract

**Introduction:**

Rice false smut (RFS), caused by *Ustilaginoidea virens* (teleomorph: *Villosiclava virens*), has emerged as a major global threat to rice production, causing reductions in yield, grain quality, and market value. Although first reported in India in the 1870s, genomic resources for this pathogen remain limited, constraining efforts toward understanding pathogen diversity and developing effective disease management strategies.

**Methods:**

In the present study, a high-quality whole-genome sequence of the Eastern Indian *U. virens* isolate NRRI-FSM-1 was generated and analyzed. Comparative whole-genome sequence (WGS) analysis was further performed using six *U. virens* strains to investigate genomic diversity, structural variation, and candidate pathogenicity-related features.

**Results:**

The assembled NRRI-FSM-1 genome was 36.3 Mb in size, comprising 985 scaffolds with an N50 of 5,781,932 bp. A total of 328,782 variants were identified, including 302,430 SNPs, 13,224 insertions, and 13,128 deletions. Additionally, 5,977 simple sequence repeats (SSRs) and 9,257 protein-coding genes were identified, representing the highest number of predicted genes reported so far among false smut genomes. Comparative genomics revealed substantial genomic diversity among the six strains, including variation in candidate effector repertoires, gene content, and population structure at both global and intra-Indian levels. Notably, significant diversity was observed among Indian strains, indicating considerable genomic variation across geographical regions.

**Discussion:**

These findings expand the pathogenomic resource base for *U. virens* in India and globally, and provide insights into genome evolution and genetic plasticity in this important rice pathogen. The generated genomic resource establishes a foundation for future studies on pathogen surveillance, virulence mechanisms, and molecular breeding strategies for rice false smut management.

## Introduction

Biotic stresses impart a significant yield penalty to rice productivity, resulting in a considerable loss in both quantity and quality (Bag et al., 2021a). Smut pathogen *Ustilaginoidea virens* Takahashi (teleomorph: *Villosiclava virens*) has increasingly become a major concern for yield and grain quality in rice. Although this pathogen was first reported from India in the 1870s (Cooke, 1878), its severity has increased in the past 15–20 years. The global prevalence of this disease is now considered alarming, as it can cause substantial reductions in grain yield and a significantly depreciate market value of the harvested grain due to contamination (Rush et al., 2000; Singh and Pophaly, 2010; Sun et al., 2020). Several factors have been implicated in the recent rise of false smut epidemics, including the large-scale cultivation of genetically uniform high-yielding rice varieties, excessive application of nitrogenous fertilizers, and changing regional and global climatic conditions that favor pathogen development (Lu et al., 2009; He et al., 2021; Sathiyaseelan et al., 2025).

Though this pathogen was first reported from India in the 1870s (Cooke, 1878), its manifestation as a major rice pathogen started in the last couple of decades. In India, false smut was a relatively minor problem decades ago, but reports since the early 2000s indicate increasing incidence and variable yield losses between 1–11% (Dodan and Singh, 1996; Mandhare et al., 2008), and localized outbreaks reaching very high incidence levels (reports up to ~85%) in certain regions (Ladhalakshmi et al., 2012). This rising prevalence underscores the urgent need for integrated management and surveillance (Pramesh et al., 2020; Zhou et al., 2024).

Rice false smut is a major disease of rice that reduces both grain yield and quality. It affects rice at the panicle stage, typically observed after the grain-filling period. The disease manifests initially as a white mycelial growth on individual grains that later manifest into smut balls, transitioning in color from yellowish-orange to green, olive-green, and finally greenish-black as they mature (Bag et al., 2021b). The global prevalence of rice false smut is alarming because it can substantially reduce grain yield and sharply depreciate market value through contamination (Rush et al., 2000; Singh and Pophaly, 2010; Zhou et al., 2024) Additionally, infected grains can contain mycotoxins (including ustiloxins and ustilaginoidins), which pose health risks to humans and livestock, and recent toxicology studies show they may cause hepatotoxicity and other adverse effects in animal models (Zhang et al., 2014; Tsukui et al., 2014; Wang et al., 2025).

Robust disease management requires deep knowledge of pathogen population composition, genetic diversity, evolutionary dynamics, virulence mechanisms, and distribution of existing and potential effector molecules. Pathogenomics; the study of genes involved in pathogenicity, effector repertoires, secondary-metabolite (toxin) biosynthetic pathways, and host–pathogen interactions is therefore central to developing durable control strategies (Kumagai et al., 2016; Zhang et al., 2024). Although the first complete *U. virens* genome (Zhang et al., 2014) and early comparative analyses revealed key components associated with pathogenicity and toxin production (Sun et al., 2013; Zhang et al., 2014), genomic resources remain geographically biased and incomplete; only a limited number of strains (mostly from China and Japan, and India) have been fully sequenced and analyzed (Wang et al., 2022; Pramesh et al., 2020; Bashyal et al., 2023; Bao et al., 2021; Xue et al., 2023).

Recent functional and comparative genomics work has advanced our understanding of *U. virens* virulence: (i) fungal effectors that suppress floral immunity and remodel host chromatin or signaling have been identified and functionally characterized, revealing mechanisms by which the fungus colonizes spikelets; (ii) regulatory pathways such as TOR signaling and certain cytochrome P450 enzymes have been implicated in coordinating development, secondary metabolism and pathogenicity; and (iii) high-quality genome assemblies and genome-wide comparisons have begun to define lineage-specific genes, effector repertoires and biosynthetic gene clusters for mycotoxins. These studies highlight both conserved virulence strategies and notable genomic diversity across isolates, stressing the need for broader sampling across rice ecologies and geographies (Yu et al., 2025; Li et al., 2025; Cao et al., 2025).

To address the gap in Indian pathogenomic data, we present here the whole genome sequence (WGS) of a virulent *U. virens* strain NRRI-FSM-1 from Eastern India, and perform comparative genome analysis with previously reported false smut genomes. Our findings expand the genomic resource base for Indian strains, help illuminate population structure and virulence gene diversity, and will support future efforts to catalog avirulence and effector genes for breeding and molecular surveillance. In the longer term, integrating genomic surveillance with climate-based risk models and toxicology data will improve forecasting and help prioritize management strategies to reduce yield loss and food-safety risk (Sathiyaseelan et al., 2025).

## Results

### Quality check and assembly of NRRI-FSM-1 strain

A whole-genome assembly of *U. virens* strain NRRI-FSM-1 was generated using the Illumina sequencing platform. The high-quality, paired reads amounted to approximately 3.97 Gb, representing ∼221-fold genome coverage, with the genome size estimated at 38 Mb (**Table 1**). Initially, 56,224,130 total reads from this strain were selected for the assembly analysis. This assembly generated a total of 10479 contigs, which were utilized in the construction of scaffolds (**Table 1**). A total of 985 scaffolds were obtained, with a combined length of 36344339 bp and an N50 value of 5,781,932 bp. Other important features of the assembly results, including N50 value, and GC content are provided in **Table 1**. The quality of the assembly was assessed using BUSCO analysis with the fungi OrthoDB version 10 dataset. This assessment resulted in 97.2% completeness with single-copy orthologs, 0.3% completeness with duplicated orthologs, 1.2% fragmented, and 1.3% missing orthologs, based on a total of 758 BUSCO groups. These 985 scaffolds were used in our subsequent analyses.

**Table 1:** Statistics of rice smut fungal genome features.

Genomic feature	NRRI-FSM-1	UV-GVT	iJS62	IPU010	P1	UV-8b
Total scaffold sequence length (bp)	36344339	26967352	38024132	33567624	38804241	37158136
Number of scaffolds	985	9157	7	139	8	8
Scaffold N50 (bp)	5781932	15534	6315893	529978	6270571	6429791
G+C content in scaffolds (%)	49.01	54.69	49.94	51.31	49.70	50.15
N content in scaffolds (%)	3.09	0.41	0.00	0.00	0.00	0.00
Number of contigs	10479	13478	7	139	9	8
Contig N50 (bp)	7561	5476	6315893	529978	6270571	6429791
Number of protein coding genes	9257	7596	7598	7522	7596	7595
Average gene length (bp)	1860	1676	1762	1755	1768	1780
Gene density (per Mb)	254.70	281.67	252.42	224.08	195.75	204.39

### Pangenome and strain specific regions in the *Ustilaginoidea virens*

A pangenome of 43 Mb was constructed using whole-genome sequences from six *Ustilaginoidea* strains: NRRI-FSM-1, UV-GVT, iJS62, IPU010, P1, and UV-8b. In this pangenome analysis, the genome sequence of UV-8b was used as the reference. The pangenome contained 8,445 genes. Presence-absence variations (PAVs) are a type of structural genetic variation in which specific DNA segments or genes are present in one strain but completely absent in others. The regions identified as PAVs in a particular strain are referred to as strain-specific regions. The PAV analysis across all strains showed that NRRI-FSM-1 had the highest number of PAVs, while another Indian strain UV-GVT had the lowest (**Table 2**). The average length of PAVs ranged from 0.81 kb in UV-GVT to 10.65 kb in iJS62, with an overall average length of 5.92 kb. Further, IPU010 contributed 18.89 Mb of consensus sequences to the pangenome, with 239 PAVs, representing the longest strain-specific region among the strains, followed by NRRI-FSM-1 with 16.62 Mb (**Table 2**). The highest number of strain-specific genes was observed in NRRI-FSM-1, while none were found in P1. NRRI-FSM-1 also had the highest strain-specific gene density (2.10 genes per Mb), significantly higher than the other strains.

**Table 2:** Pangenome summary.

Strain	Genome size (Mb)	Total PAVs	PAVs included in pangenome	PAVs’ mean length (kb)	Strain-specific sequences (Mb)	Strain-specific genes	Strain-specific gene density (per Mb)
NRRI-FSM-1	36.34	2671	318	2.43	16.62	35	2.10
UV-GVT	26.96	308	61	0.81	7.53	3	0.39
iJS62	38.02	587	260	10.56	6.13	2	0.32
IPU010	33.56	704	239	5.38	18.89	5	0.26
P1	38.80	523	150	10.45	5.69	0	0.00

### Synteny of NRRI-FSM-1 with reference genome

**Figure 1:**
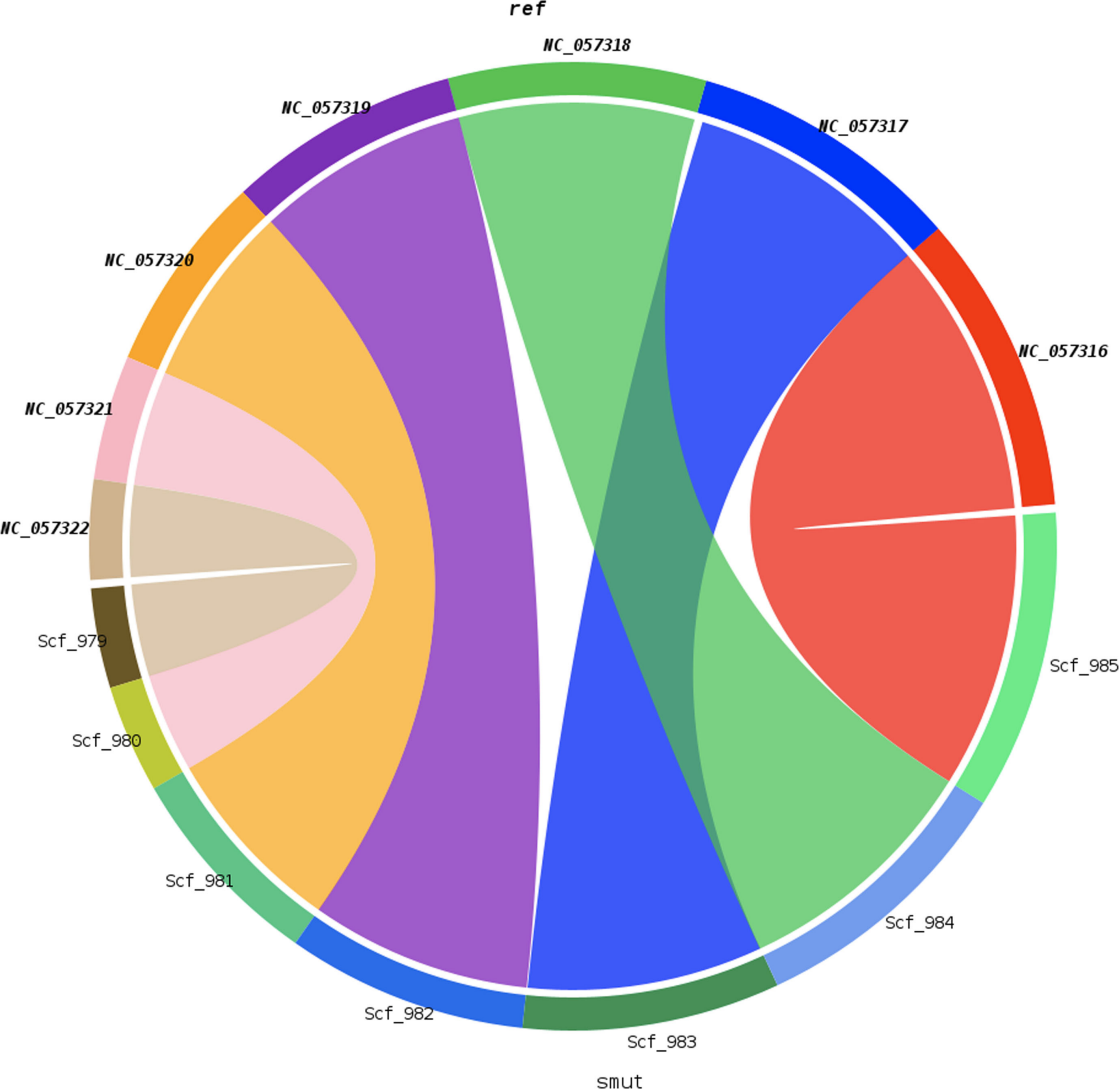
Synteny between NRRI-FSM-1 scaffolds and UV-8b reference chromosomes. The circular chord diagram illustrates syntenic relationships between the query genome scaffolds (Scf_979-Scf_985) of *Ustilaginoidea virens* strain NRRI-FSM-1 (“smut”) and the reference chromosomes (NC_057316-NC_057322) of *U. virens* strain UV-8b (“ref”), as generated by SyMAP. Each arc segment represents an individual scaffold or chromosome, with colored ribbons connecting homologous regions across the two genomes. The diagram highlights conserved genomic blocks and structural alignments, providing a comparative view of genome organization between the query and reference assemblies.

**Figure 2:**
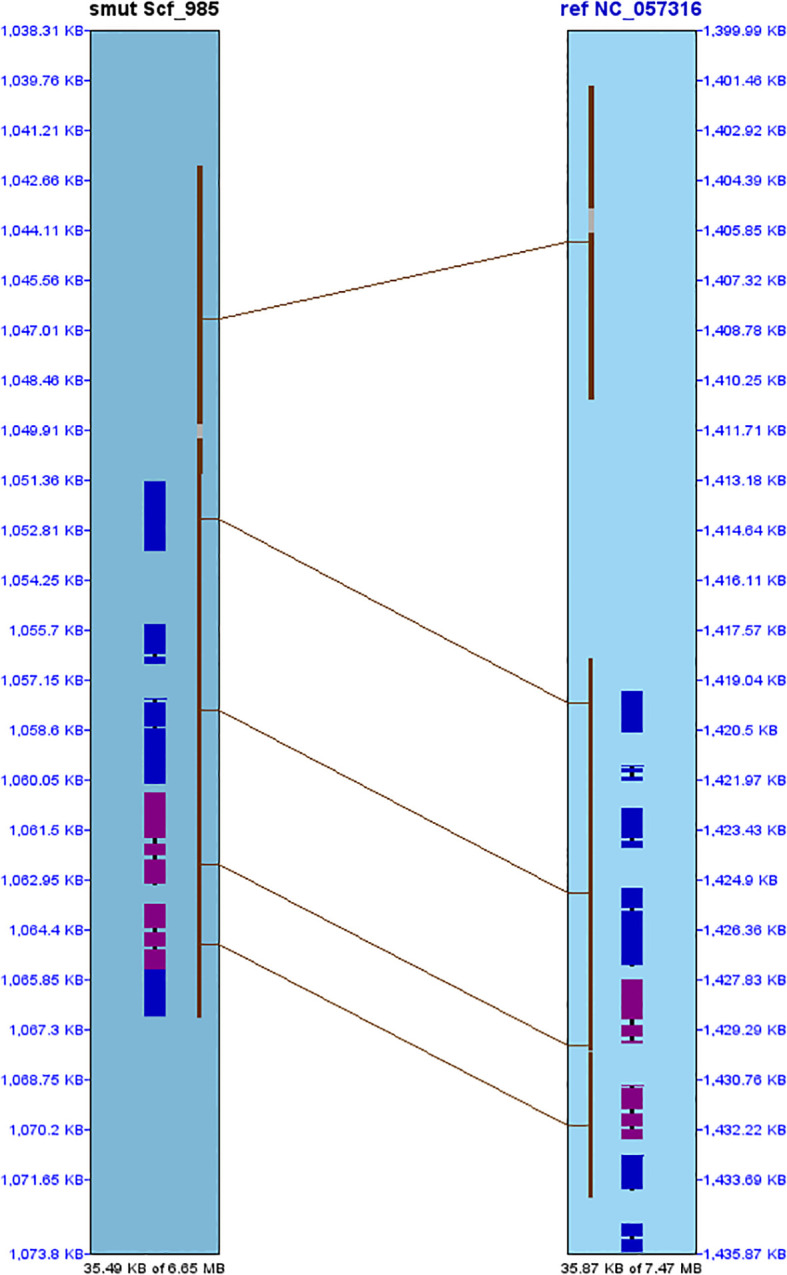
Pairwise synteny comparison between chromosome 1 from NRRI-FSM-1 and UV-8b. The synteny plot illustrates conserved gene order and collinearity between scaffold Scf_985 (left) and chromosome NC_057316 (right). Blue and pink blocks represent exons in + and – orientations, respectively, while brown lines denote syntenic links between homologous regions.

A comparison of the mapped regions between NRRI-FSM-1 and UV-8b was performed to determine the order of short nucleotide sequences and the level of similarity between them. A dot plot of all seven chromosomes was used to compare the NRRI-FSM-1 and UV-8b genomes (**Figure 1**). A total of 7,737 hits were obtained in this synteny analysis, with Pearson correlation coefficient (Pearson R) values for all hits exceeding 0.99. Pearson R, which measures the linear association between two variables, ranges from +1 to -1. Of the 7,737 hits, 1,589 were matched to chromosome 1. Additionally, two short regions, 35.87 kb and 35.49 kb from chromosome 1 of UV-8b and NRRI-FSM-1, respectively, were separately aligned to provide a clear view of matching DNA fragments and their order between these two genomes (**Figure 2**). The remaining hits were distributed across rest of the six chromosomes (2 to 7), with a maximum of 1,513 hits on chromosome 2 and a minimum of 509 hits on chromosome 6.

### Strain specific SNPs and InDels in NRRI-FSM-1

**Figure 3:**
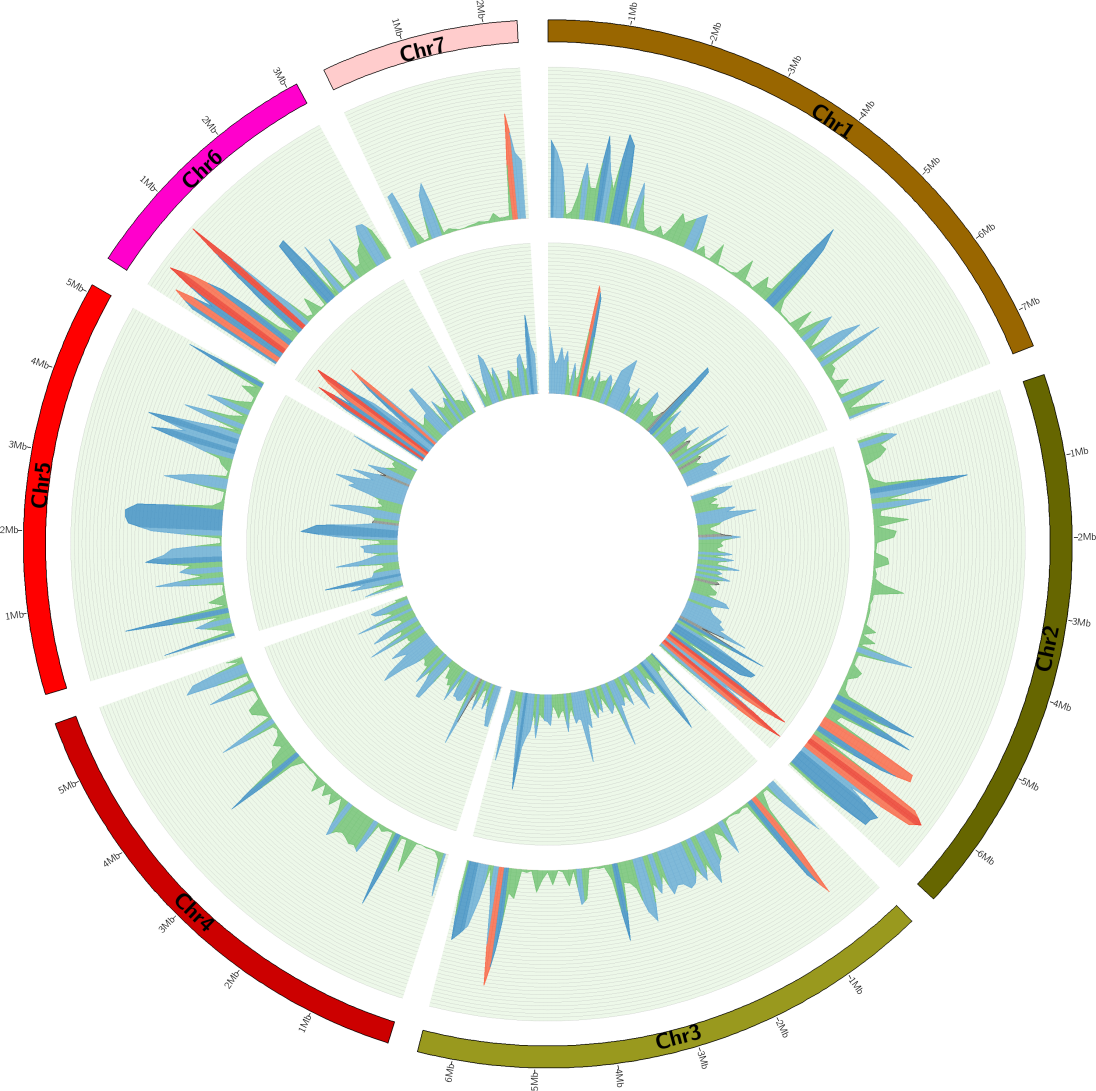
Genome organization of NRRI-FSM-1. The size of each chromosome is marked in Mb. From the outer to the inner circles, the plot displays chromosomes, SNPs, and InDels, respectively. SNP and InDel densities are shown as line diagrams per 100 kb genomic region and scaled by percentage. Regions filled with dark red, red, dark blue, blue, and green indicate SNP density values greater than 4, 3, 2, 1, and less than 1, respectively. The same color scheme applies to InDel density values greater than 0.25, 0.2, 0.15, 0.05, and less than 0.05, respectively.

Small variations in the NRRI-FSM-1 genome were determined by following a variant calling pipeline using UV-8b genome as reference. Overall, 85.81% the processed reads (48,903,627 quality-passed reads) of NRRI-FSM-1 were successfully mapped to the reference sequences. Only 0.11% duplication was detected among the mapped reads. In the small variant analysis, a total of 302,430 single nucleotide polymorphisms (SNPs), and InDels comprising 13,224 insertions and 13,128 deletions were identified in the in the NRRI-FSM-1 (**Table 3**). The variant rate was calculated for each chromosome, defined as the number of variants per effective genomic length, wherein the effective genomic length is the total length of the variant-containing chromosomes in bases. A total of 328,782 variants, comprising SNPs and InDels were detected in the NRRI-FSM-1 genome, with an overall variant rate of 112 (**Table 3**). Further, chromosome 1 had the highest variant rate of 185, while chromosome 6 had the lowest rate at 69. This indicates that chromosome 6 of NRRI-FSM-1 showed the highest similarity with the corresponding chromosome from the reference strain genome, UV-8b, when observing the chromosome-wise distribution of variants. The density of SNPs and InDels (per 100 kb of genomic regions) was estimated for each chromosome and plotted on a circular graph (**Figure 3**).

**Table 3:** Distribution of variants identified in NRRI stain and its variants rate detail.

Chromosome	Length	Variants	Variants rate
NC_057316.1	7,466,040	56,013	133
NC_057317.1	6,775,769	64,704	104
NC_057318.1	6,429,791	60,736	105
NC_057319.1	5,747,404	31,000	185
NC_057320.1	5,103,626	55,378	92
NC_057321.1	3,124,897	44,759	69
NC_057322.1	2,412,559	16,192	148
Total	37,060,086	328,782	112

### Comparative structural and functional features in *U. virens* isolates

**Figure 4:**
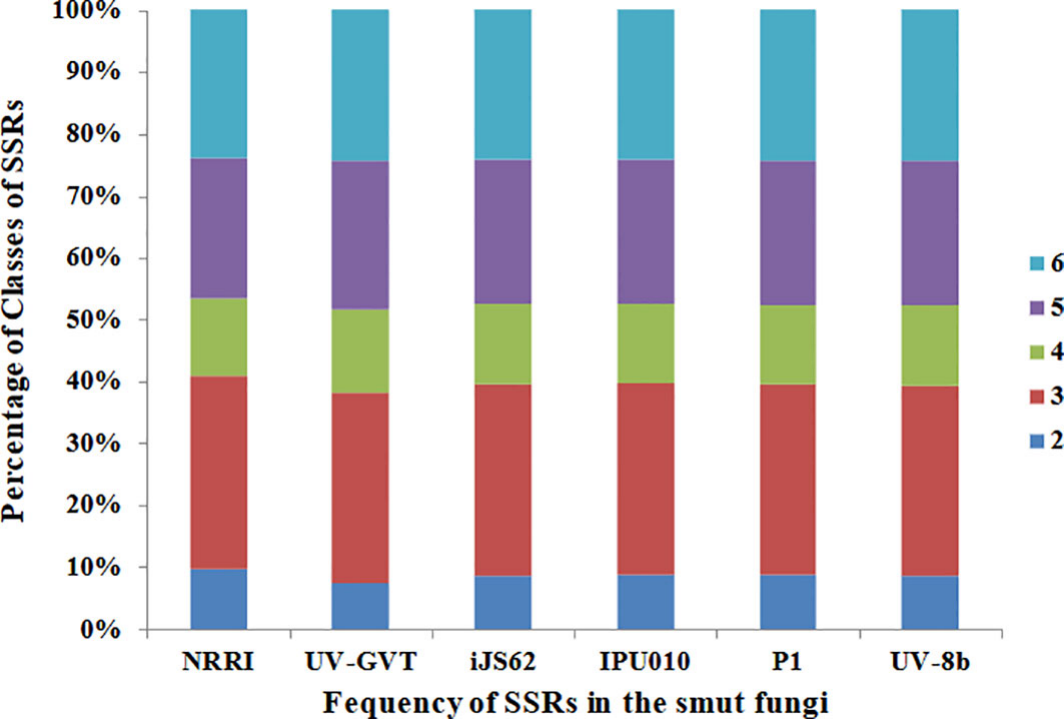
Distribution of simple sequence repeats (SSRs) across six *U. virens* genomes. The stacked bar chart represents the percentage distribution of different SSR motif classes (di-, tri-, tetra-, penta-, and hexa-nucleotide repeats) in NRRI-FSM-1, UV-GVT, iJS62, IPU010, P1, and UV-8b genomes.

**Figure 5:**
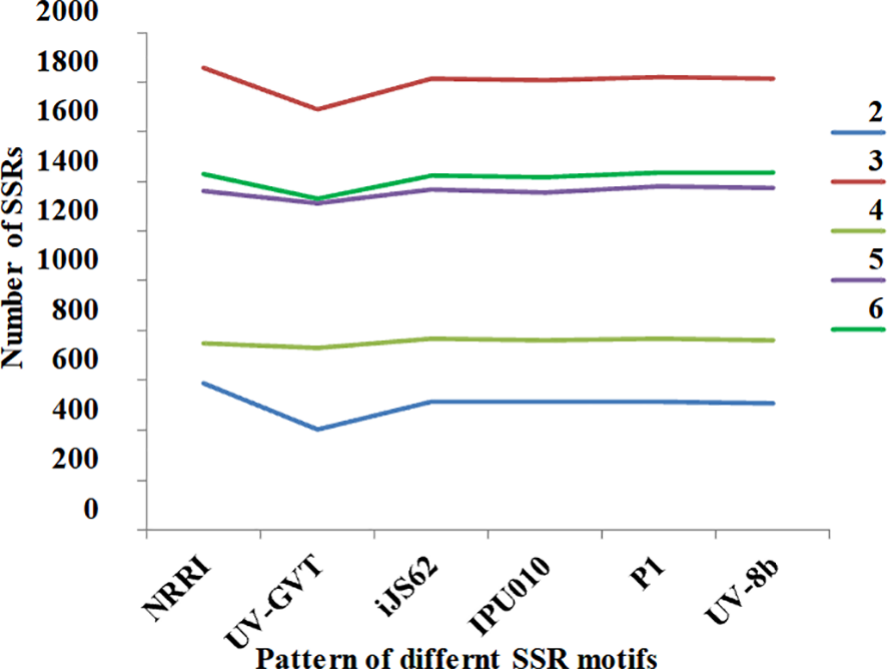
Distribution of SSR motifs among all six *U. virens* genomes. This graph shows the frequency of SSRs, categorized by motif types (2–6), across the six genomes, NRRI-FSM-1, UV-GVT, iJS62, IPU010, P1, and UV-8b.

For structural genomics, various repeats such as simple sequence repeats (SSRs) and transposable elements were analyzed in all six strains of the rice smut fungus *U. virens*. A total of 5,977 SSRs were identified in the NRRI-FSM-1 genome sequences (**Table 4**). Of these, the highest proportion (31%) consisted of tri-nucleotide SSR motifs, followed by hexa-, penta-, tetra-, and di-nucleotide motifs (**Figure 4**). A similar trend of SSR motif distribution was observed in the remaining strains (**Figure 5**). Overall, a total of 34,969 SSRs were detected across all six strains. The highest percentage of SSRs (17.09%) was found in NRRI-FSM-1, while the lowest percentage (15.61%) was observed in UV-GVT (**Table 4**).

**Table 4:** Distribution of different classes of SSR.

SSR Class	Frequency of SSR classes in the smut fungi						
	NRRI-FSM-1	UV-GVT	iJS62	IPU010	P1	UV-8b	Total
Di-nt.	586	404	511	516	515	510	3042
Tri-nt.	1853	1686	1812	1806	1819	1812	10788
Tera-nt.	749	729	769	759	764	763	4533
Penta-nt.	1361	1310	1364	1354	1376	1372	8137
Hexa-nt.	1428	1330	1424	1417	1437	1433	8469
Total	5977	5459	5880	5852	5911	5890	34969

For understanding the genomic structure of the rice smut fungi in a better way, transposable repeat elements were identified in strain NRRI-FSM-1, and a total of 15,268 interspersed repeats, including 12,906 classified and 2,362 unclassified, were obtained in its genome (**Table 5**). The identified repeats were classified based on the repeat library databases; Repbase and EDTA. In total, 26.52% of the NRRI-FSM-1 genomic sequences were masked as repeat elements. Similarly, the genome sequences of the other five *U. virens* strains, including the reference strain UV-8b, were also subjected to repeat masking. The highest level of repeats was masked in the P1 genome (32.14%), while the lowest level was detected in UV-GVT genome (7.94%) (**Table 5**).

**Table 5:** Copy number variation of different repeat classes in the smut fungal genome.

Major repeat class/family	NRRI-FSM-1	UV-GVT	iJS62	IPU010	P1	UV-8b
Retroelements (REs) (SINEs, LINEs, LTRs)	6098 (15.98%)^*^	5838 (4.64%)	6819 (18.22%)	5032 (13.28%)	7185 (18.73%)	6514 (17.26%)
Total SINEs	31 (0.01%)	29 (0.01%)	30 (0.01%)	30 (0.01%)	30 (0.01%)	30 (0.01%)
Penelope (a class of SINE)	11 (0.00%)	0 (0.00%)	8 (0.01%)	6 (0.00%)	13 (0.00%)	15 (0.00%)
Total LINEs	373 (0.27%)	187 (0.13%)	426 (0.40%)	363 (0.37%)	458 (0.43%)	390 (0.36%)
CRE/SLACS (a class of LINE)	4 (0.00%)	3 (0.00%)	7 (0.00%)	3 (0.00%)	6 (0.00%)	4 (0.00%)
Total LTRs	5694 (15.7%)	5622 (4.00%)	6363 (17.81%)	4639 (12.89%)	6697 (18.29%)	6094 (16.89%)
Ty1/Copia (a class of LTR)	180 (0.06%)	142 (0.04%)	165 (0.04%)	153 (0.05%)	181 (0.04%)	170 (0.05%)
Gypsy/DIRS1 (a class of LTR)	205 (0.06%)	295 (0.07%)	201 (0.06%)	203 (0.06%)	210 (0.06%)	201 (0.06%)
Total DNA transposons (DTs)	6808 (8.82%)	3341 (2.8%)	6804 (10.66%)	5413 (8.73%)	7075 (10.86%)	6633 (10.58%)
Hobo-Activator (a class DT)	5 (0.00%)	13 (0.00%)	6 (0.00%)	5 (0.00%)	8 (0.00%)	7 (0.00%)
Tc1-IS630-Pogo (a class of DT)	54 (0.01%)	73 (0.02%)	46 (0.01%)	50 (0.01%)	52 (0.01%)	49 (0.01%)
Tourist/Harbinger (a class of DT)	17 (0.01%)	17 (0.00%)	28 (0.02%)	19 (0.01%)	32 (0.02%)	23 (0.01%)
Unclassified repeats (URs)	2362 (1.71%)	1045 (0.05%)	3418 (2.41%)	2762 (2.20%)	3699 (2.55%)	3364 (2.45%)
Total interspersed repeats (REs, DTs, URs)	15268 (26.52%)	10224 (7.94%)	17041 (31.29%)	13207 (24.21%)	17959 (32.14%)	16411 (30.29%)

SINEs, Short interspersed nuclear elements; LINEs, Long interspersed nuclear elements; LTRs, Long terminal repeats, ^*^Percentage of respective genome sequences.

Further, the maximum copy numbers of retro-elements were identified as long terminal repeats (LTRs) across all six strains, and the number varied from 4,639 in IPU010 to 6,697 in P1. However, the copy number of short interspersed nuclear elements (SINEs) was highly conserved across these strains, with a minimum of 29 in UV-GVT and a maximum of 31 in NRRI-FSM-1. On the contrary, there was a significant variation in the number of long interspersed nuclear elements (LINEs) in these strains with highest and lowest number observed in P1 (458 LINEs) and UV-GVT (187 LINEs), respectively. Similarly, for retro-elements, the highest numbers of DNA transposons (DTs) were observed in P1 (7,075 DTs), and the lowest numbers were detected in UV-GVT (3,341 DTs). The occurrence of unclassified repeats (URs) was highest in P1, followed by UV-8b, iJS62, IPU010, and UV-GVT (**Table 5**).

**Figure 6:**
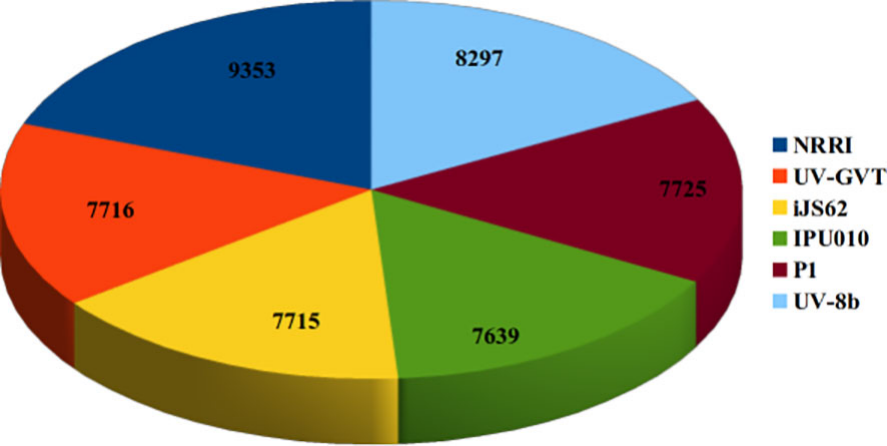
Summary of predicted genes in six *U. virens* genomic datasets. The 3D pie chart illustrates the total number of predicted genes in the genomes of NRRI-FSM-1, UV-GVT, iJS62, IPU010, P1, and UV-8b. The proportional distribution highlights dataset-specific variations in gene prediction.

Finally, the assembled sequences of all six *U. virens* strains were subjected to gene prediction, and the highest number of genes predicted from NRRI-FSM-1 (9353), followed by UV-8b, IPU010, P1, UV-GVT, and iJS62 (**Figure 6**). The predicted gene sequences were then used for functional annotation.

### Comparative phylogenetic study of *Ustilaginoidea virens* strains

**Figure 7:**
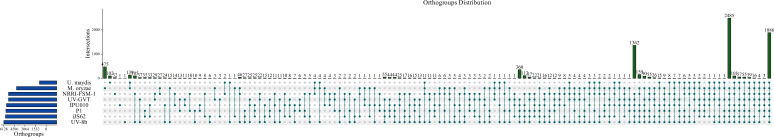
Orthogroup distribution across eight fungal genomes. The UpSet plot shows the number of orthogroups identified in *Ustilago maydis*, *Magnaporthe oryzae*, and six *Ustilaginoidea virens* strains (NRRI-FSM-1, UV-GVT, iJS62, IPU010, P1, and UV-8b). Blue horizontal bars indicate the total orthogroups per genome, while green vertical bars represent intersections, highlighting shared orthogroups among different genome combinations.

**Figure 8:**
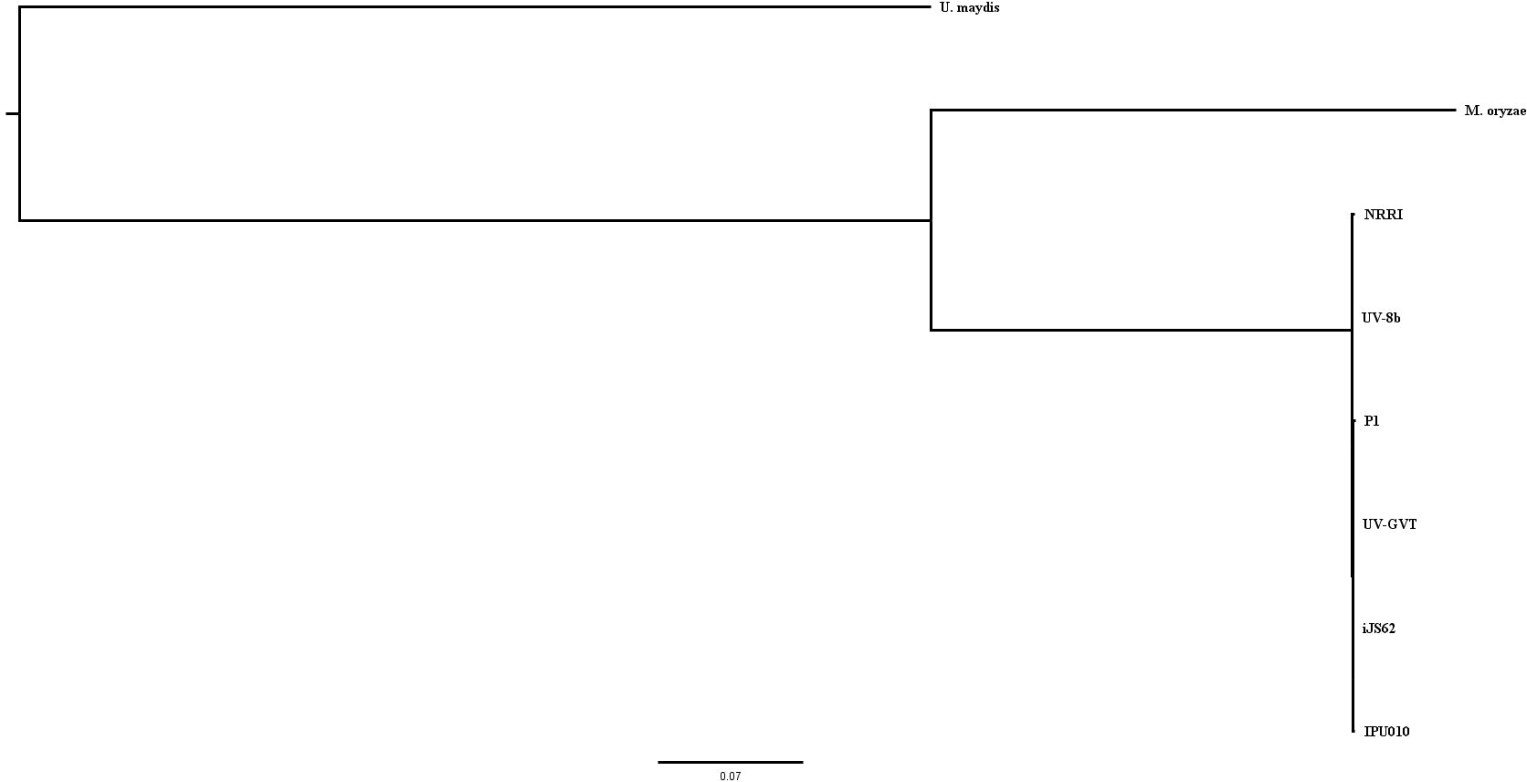
Phylogenomic analysis based on single-copy orthogroups. This rooted phylogenetic tree depicts the evolutionary relationships among eight genomes, *U. maydis*, *M. oryzae*, and six *U. virens* strains (NRRI-FSM-1, UV-GVT, iJS62, IPU010, P1, and UV-8b), based on conserved single-copy orthogroups. The branching topology and genetic distance scale (0.07) reflect divergence levels, with closer branches indicating higher sequence similarity and shared ancestry. All *U. virens* strains cluster together as a major group, whereas *M. oryzae* and *U. maydis* serve as outgroups.

For the comparative genome study, a total of eight genomes were used in the phylogenomic analysis: six *Ustilaginoidea* strains (UV-GVT, iJS62, IPU010, NRRI-FSM-1, P1, and UV-8b) and two outgroup genome sequences (*Magnaporthe oryzae* and *Ustilago maydis*). A total of 68,434 protein sequences from all eight genomes were used as an input data for the identification of orthogroups and single-copy orthogroups among these genomes. Over 80% of genes were found to be present in the identified orthogroups (**Table 6**). Approximately 7% of orthogroups were identified as genome-specific. Additionally, only 16.77% (1,474) of orthogroups were classified as single-copy orthogroups, while the rest (83.23% of the total 8,790 orthogroups) contained duplicated genes. The genome-wise distribution of orthogroups is shown in Figure (**Figure 7**). In the phylogenetic tree, all six *U. virens* strains were highly conserved and clustered together into a single group. As expected, both outgroup genomes (*Ustilago maydis* and *Magnaporthe oryzae*) did not form any sister taxa with *U. virens* strains (**Figure 8**). It was observed that *U. maydis* was most distantly related to the *Ustilaginoidea* strains compared to *M. oryzae* relatedness to these *Ustilaginoidea* strains.

**Table 6:** Summary of orthologous groups analysis.

Feature of orthogroups	Value
Number of genomes	8
Number of genes	68434
Number of genes in orthogroups	55077
Number of unassigned genes	13357
Percentage of genes in orthogroups	80.5
Percentage of unassigned genes	19.5
Number of orthogroups	8790
Number of genome-specific orthogroups	611
Number of genes in genome-specific orthogroups	1762
Percentage of genes in genome-specific orthogroups	2.6

### Identification of secondary metabolites from *Ustilaginoidea virens*

**Figure 9:**
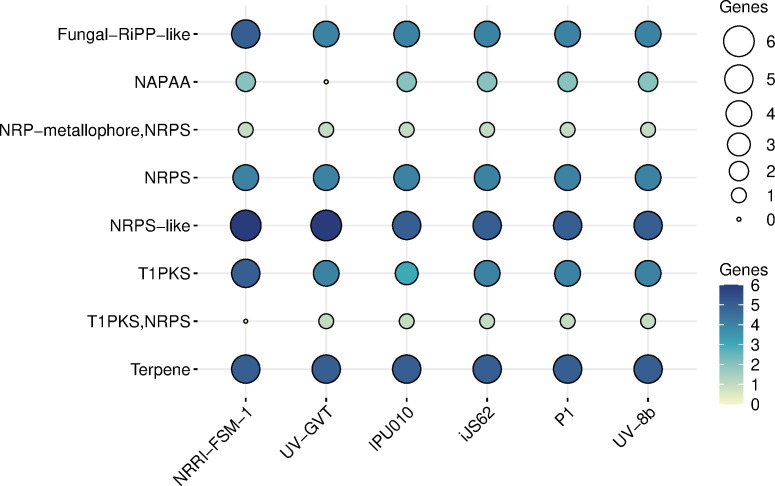
Secondary metabolite biosynthetic gene clusters in six *Ustilaginoidea virens* strains. Bubble plot summarizing secondary metabolite biosynthetic gene clusters (BGCs) predicted by antiSMASH in six rice smut fungi (*U. virens* strains NRRI-FSM-1, UV-GVT, iJS62, IPU010, P1, and UV-8b). Rows represent distinct BGC types, while columns correspond to individual strains. Bubble size and color intensity indicate the number of genes per cluster type, highlighting variation in BGC abundance across strains.

To identify secondary metabolites, we performed antiSMASH analysis separately for each strain, including the reference strain UV-8b. In this analysis, various classes of secondary metabolites were identified using the ‘relaxed’ parameter setting for strictness. A total of 156 genes from all six strains were found to encode enzymes responsible for the synthesis of different classes of secondary metabolites (**Table 7**). All secondary metabolites identified from NRRI-FSM-1 are presented graphically (**Figure 9**). Similar graphical representations were generated as results for each strain in the antiSMASH analysis. These enzymes include canonical classes such as polyketide synthases (PKSs), non-ribosomal peptide synthetases (NRPSs), as well as Fungal-RiPP-like, NAPAA, and Terpene. The number of predicted core biosynthetic or backbone genes in all six strains, relative to the total genes, aligns with the prolific secondary metabolite production of rice false smut fungi (**Table 7**), which typically contain around 25 to 28 backbone genes per strain. Interestingly, NRRI-FSM-1 surpasses the other *Ustilaginoidea* strains by few additional backbone genes.

**Table 7:** Number of identified secondary metabolites in the smut fungi.

Secondary metabolites	NRRI-FSM-1	UV-GVT	IPU010	iJS62	P1	UV-8b
Fungal-RiPP-like	5	4	4	4	4	4
NAPAA	2	0	2	2	2	2
NRP-metallophore, NRPS	1	1	1	1	1	1
NRPS	4	4	4	4	4	4
NRPS-like	6	6	5	5	5	5
T1PKS	5	4	3	4	4	4
T1PKS,NRPS	0	1	1	1	1	1
Terpene	5	5	5	5	5	5
Total	28	25	25	26	26	26

### Family of CAZymes in *Ustilaginoidea virens*

**Figure 10:**
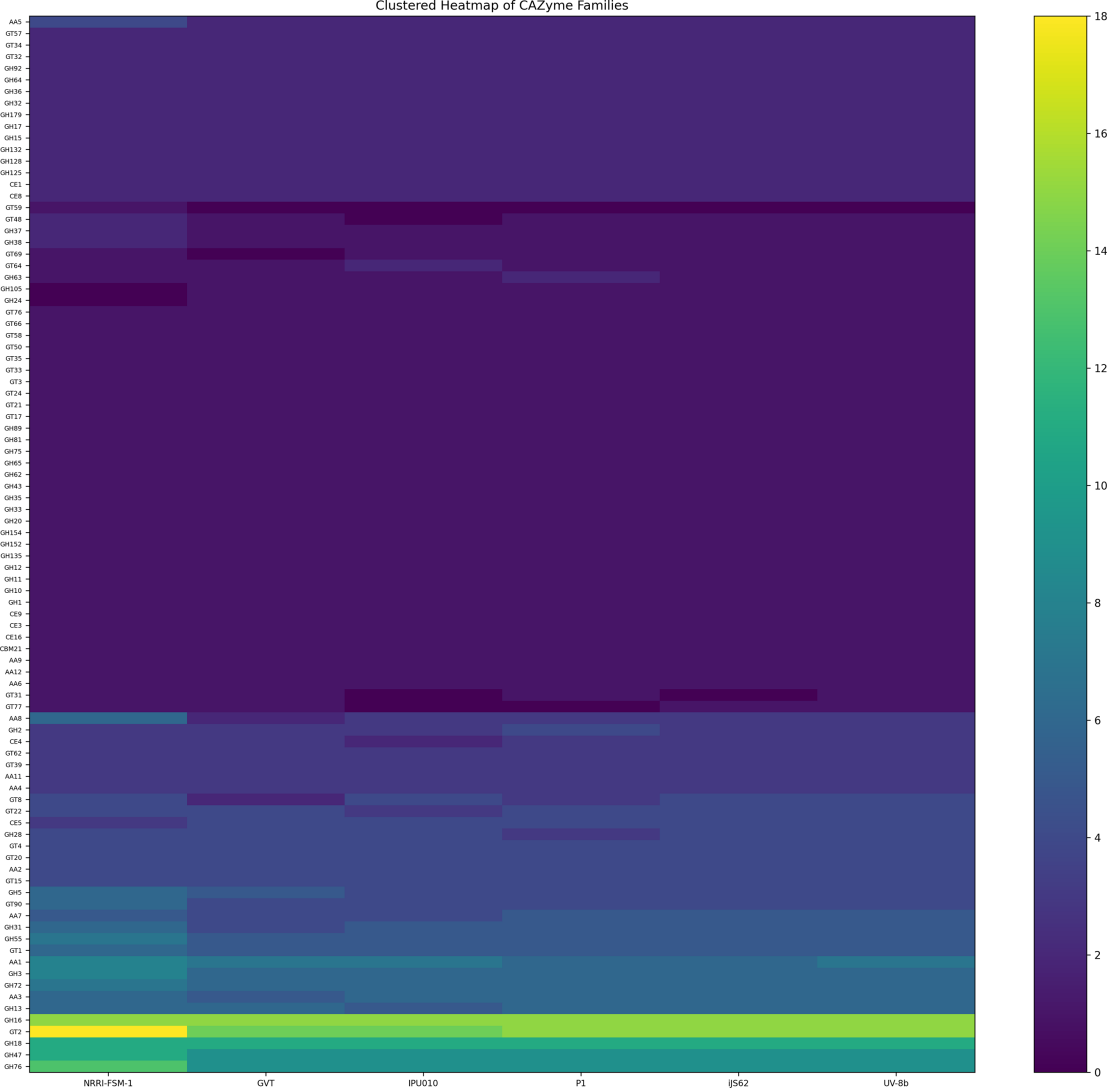
CAZyme family distribution in six *Ustilaginoidea virens* strains. Clustered heatmap showing the distribution of carbohydrate-active enzyme (CAZyme) families predicted in six rice smut fungi (*U. virens* strains NRRI-FSM-1, UV-GVT, iJS62, IPU010, P1, and UV-8b). Rows represent individual CAZyme families, while columns correspond to the fungal strains. Color intensity reflects the number of genes per family, highlighting variation in CAZyme repertoires across strains.

CAZyme identification revealed the presence of 273 different CAZyme families in NRRI-FSM-1 (**Figure 10**; **Table 8**). Further analysis showed that the most abundant family was glycoside hydrolase (GH; 138 GH families). Following GH, glycosyltransferases (GT) were the second-most abundant family, with 79 GT families. The third-largest family was auxiliary activities (AA), comprising 42 AA families. Interestingly, the carbohydrate-binding module family 21 (CBM21), which is known for its granular starch-binding function (sometimes referred to as starch-binding domains, SBD), was the least abundant in the CAZyme family composition (**Table 8**).

**Table 8:** Comparative CAZymes family profile.

Family	NRRI-FSM-1	GVT	IJS62	IPU010	P1	UV-8B	Total
AA	42	33	35	35	35	36	216
CBM	1	1	1	1	1	1	6
CE	13	14	14	13	14	14	82
GH	138	124	124	123	125	124	758
GT	79	67	70	67	69	71	423
Total	273	239	244	239	244	246	1485

Downstream analysis revealed that the GT2 family was the most abundant, followed by GH16, GH18, GH76, GH47, and AA1 (**Supplementary Table 1**). Other highly abundant families, such as GH3, GH72, AA3, GH13, GH55, and GT1 were equally present across the studied strains. In total, 1,485 CAZymes were detected in all six *Ustilaginoidea* strains, including NRRI-FSM-1. A comparison of CAZymes across all strains revealed that NRRI-FSM-1 had approximately 2% more families (18.38%) than any other strain. Additionally, the GT59 family was uniquely present in NRRI-FSM-1, while the GH24 family was absent in this strain.

### Candidate effectors from *Ustilaginoidea virens*

**Figure 11:**
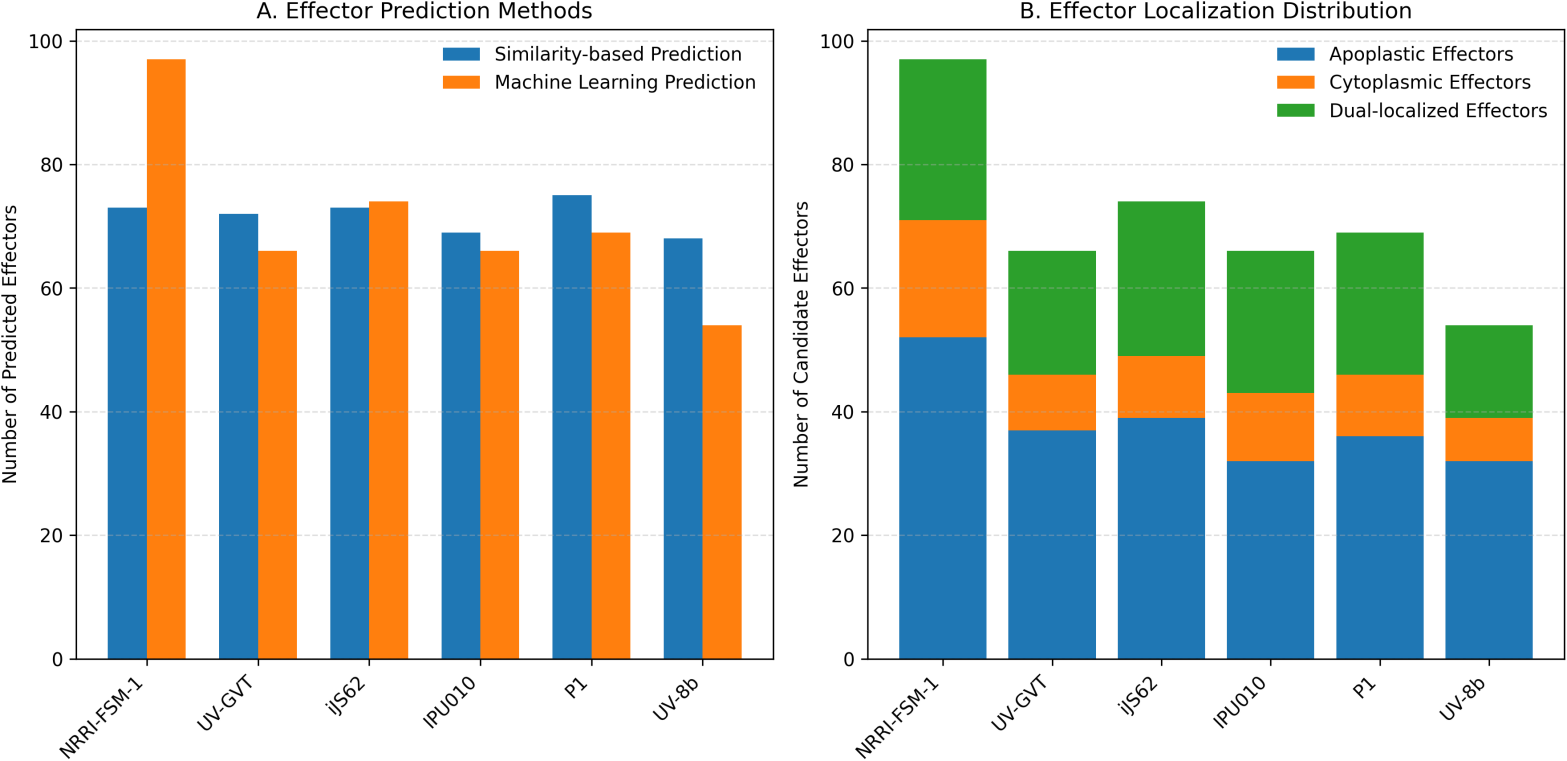
Identification of effectors and their localization in rice smut fungi. **(A)** shows the number of predicted effectors in each strain based on similarity-based and machine learning approaches. **(B)** displays the distribution of candidate effectors across apoplastic, cytoplasmic, and dual-localization categories. These plots are based on summarized effector prediction data from six *Ustilaginoidea virens* strains, NRRI-FSM-1, UV-GVT, iJS62, IPU010, P1, and UV-8b.

For the identification of candidate effectors, secretome analysis was performed to detect putative candidate effector molecules from all six genomes: NRRI-FSM-1, UV-8b, IPU010, P1, UV-GVT, and iJS62. A similar pattern was observed across each step of this analysis (**Figure 11**), with the number of proteins obtained in the refined secretome analysis being nearly identical among the six genomes (**Table 9**, **Supplementary Figure 1**). Furthermore, N-terminal domains, signal peptide (SP), chloroplast transit peptide (cTP), thylakoid luminal transit peptide (lTP), and mitochondrial transit peptide (mTP), were predicted. The variation in their proportions across the genomes was minimal, with only a 0.42% difference between the maximum (8.77% in iJS62) and the minimum (8.35% in NRRI-FSM-1). Similarly, for signal peptide–containing proteins, a deviation of 0.52% was observed, ranging from 7.64% in IPU010 to 7.12% in NRRI-FSM-1, when analyzed with SignalP to verify the trend from maximum to minimum values. In the same manner, differences of 0.43%, 0.98%, and 0.46% were recorded for predictions of transmembrane helices, homology-based subcellular localizations, and amino acid composition–based subcellular localizations, respectively (**Supplementary Figure 1**). The refined secretome datasets for each strain were then analyzed separately to identify candidate effectors using two approaches, machine learning–based prediction and similarity search–based detection with known effector sequences (**Table 10**). Overall, less than 39% of the refined secretome was identified as candidate effectors, with the maximum being 38.65% in NRRI-FSM-1. This proportion increased by approximately 3% when similarity searches were performed against known effector databases, DFVF (Database of Known Fungal Virulence Factors) and PHI-base, resulting in a maximum of 41.21% in UV-8b. A total of 9,686 known effector sequences were used for the similarity search analysis. Comparison of results from both methods revealed the highest number of common effectors (17) in NRRI-FSM-1, followed by 14, 13, 12, 12, and 11 in iJS62, UV-GVT, P1, UV-8b, and IPU010, respectively. The machine learning–based prediction method also classified the candidate effectors according to their cellular localization into apoplastic effectors, cytoplasmic effectors, and undetermined/both apoplastic–cytoplasmic effectors. On average, 53.72% of effectors were predicted as apoplastic, 31.14% as both types, and 15.14% as cytoplasmic (**Table 10**). Lists of candidate effectors identified by the machine learning method are provided in **Supplementary Table 2**. The known effectors matched for NRRI-FSM-1 are listed in **Supplementary Table 3**. In the identification of known effectors, a total of five effectors, UvHrip1, Cut1, Uv1809, UvCBP1, and SCRE2, belonged to *U. virens*. All these effectors were present in UV-GVT, while, except for SCRE2, the remaining four were also found in NRRI-FSM-1. However, only two of these effectors, UvHrip1 and Cut1, were detected in iJS62, IPU010, P1, and UV-8b.

**Table 9:** Identification of refine secreted proteins from the smut fungi.

Strain	Total proteins	TargetP	SignalP	TMHMM	ProtComp	WolfPsort
NRRI-FSM-1	10056	840	716	575	341	251
GVT	7596	647	549	410	228	186
IJS62	7598	666	578	437	233	198
IPU010	7522	659	575	438	217	185
P1	7596	663	572	430	244	200
UV-8B	7595	655	569	427	183	165

**Table 10:** Summary of potential candidate effectors.

Strain	Prediction by similarity search	Prediction by machine leaning method			
		Candidate effectors	Apoplastic effectors	Cytoplasmic effectors	Apoplastic/ Cytoplasmic effectors
NRRI-FSM-1	73	97	52	19	26
GVT	72	66	37	9	20
IJS62	73	74	39	10	25
IPU010	69	66	32	11	23
P1	75	69	36	10	23
UV-8B	68	54	32	7	15

## Discussion

In rice, smut pathogen *Ustilaginoidea virens* has increasingly become a major concern for yield and grain quality. The global prevalence of this disease is now considered alarming, as it can cause substantial reductions in grain yield and a significantly depreciate market value of the harvested grain due to contamination (Sun et al., 2020). In this context, deciphering the pathogenomics of *U. virens*, including its genome structure, and its comparative genomic features, is critical for understanding population structure, genetic diversity, and evolutionary dynamics across regional and global pathogen populations to devise effective strategies for the management of this pathogen in rice.

### Pangenome and strain specific analysis reveal precise regions of the rice smut fungi

The present study generated high-quality paired-end sequence reads for *U. virens* strain NRRI-FSM-1, with an estimated genome size of approximately 38 Mb, consistent with previously reported genomes (Xue et al., 2023). Pangenome analysis incorporating whole-genome sequences from six *Ustilaginoidea* strains (NRRI-FSM-1, UV-GVT, iJS62, IPU010, P1, and UV-8b) resulted in a total pangenome of 43 Mb. The larger size of the pangenome compared with individual genome assemblies likely reflects the presence of a substantial accessory gene repertoire across these strains (He et al., 2025). Among the analyzed isolates, NRRI-FSM-1 exhibited the highest number of presence–absence variations (PAVs), whereas the Indian isolate UV-GVT showed the lowest. This observation highlights notable genomic variation even among Indian strains, suggesting potential regional diversification. Such variation in the PAVs may reflect local adaptation processes operating within pathogen populations (Joubert and Krasileva, 2024). Consistent with this, NRRI-FSM-1 also harbored the highest number of strain-specific genes and displayed the greatest strain-specific gene density (2.10 genes per Mb). Higher strain-specific gene density may indicate genomic regions potentially associated with adaptive evolution in plant pathogens (Zaccaron and Stergiopoulos, 2024), however this is remains a testable hypothesis in *U. virens*.

### NRRI-FSM-1 harbor high rate small nucleotide variations

The small sequence variation in the form of single nucleotide polymorphisms (SNPs) and insertions–deletions (InDels) was assessed in the NRRI-FSM-1 genome using the reference genome UV-8b. A total of 302,430 SNPs, and InDels comprising 13,224 insertions, and 13,128 deletions were detected. In total, 328,782 variants, comprising SNPs and InDels were detected in the NRRI-FSM1 genome, corresponding to an overall variant rate of 112 per Mb. Among the chromosomes, chromosome 1 exhibited the highest variant rate (185), whereas chromosome 6 showed the lowest rate (69). Elevated nucleotide diversity may reflect evolutionary divergence among strains and potential adaptation to regional agroecosystems. Such genome-wide polymorphisms can serve as useful markers for future studies on population structure, genetic diversity, and evolutionary dynamics of *U. virens* (Ye et al., 2016).

### Structural and functional genomics of *U. virens* reveals significant genomic variation

Simple sequence repeats (SSRs) and transposable elements (TEs) were analyzed as key structural components across six *U. virens* genomes. A total of 5,977 SSRs were identified in the NRRI-FSM-1, which was higher than the other isolates. NRRI-FSM-1 also exhibited the highest proportion of SSRs (17.09%), whereas the Indian isolate UV-GVT had the lowest proportion (15.61%). These findings indicate notable structural genomic variation among *U. virens* strains, including those originating from India. Microsatellites or SSR motifs are well-established structural components of fungal genomes and serve as useful resource for the development molecular marker in studies of genetic diversity and population structure (Groppe et al., 1995; Bucheli et al., 2000).

Transposable elements are ubiquitous components of eukaryotic genomes, including those of fungal plant pathogens, and play important roles in shaping genome plasticity through chromosomal rearrangements, gene duplication or deletion, and modulation of gene expression. In the present study, 15,268 interspersed repeats were in the NRRI-FSM-1 genome, accounting for 26.52% of its total genome size. Among the six *U. virens* strains analyzed, P1 contained the highest proportion of interspersed repeats (32.14%), whereas the lowest proportion was observed in the Indian strain UV-GVT (7.94%). No significant variation in SINE elements was observed among the strains, however, substantial variation was detected in the number of LINE elements, with the highest abundance in P1 and lowest in UV-GVT, respectively. Notably, NRRI-FSM-1 contained a considerably higher number of LINE elements (373) compared with UV-GVT, suggesting differences in repeat-mediated genome evolution among these isolates. Such variation in repeat content between the two Indian strains highlights the genomic heterogeneity and possible evolutionary divergence within regional *U. virens* populations. Consistent with these structural differences, NRRI-FSM-1 also exhibited the highest number of predicted genes among the six strains, whereas UV-GVT contained the lowest number (7,716). The higher gene count observed in NRRI-FSM-1 strain, compared to those predicted in UV-GVT, may reflect underlying genomic variability; however, differences in genome assembly and annotation pipelines between studies may also contribute to this variation.

### Comparative genome study of *Ustilaginoidea virens* strains

A comparative phylogenomic analysis was conducted six *Ustilaginoidea* strains (UV-GVT, iJS62, IPU010, NRRI, P1, and UV-8b), along with two outgroup genomes, *M. oryzae* and *U. maydis*. Orthogroup analysis revealed that 16.77% (1,474) of the identified orthogroups were classified as single-copy orthogroups, whereas the remaining 83.23% of the total 8,790 orthogroups contained duplicated genes. Gene duplication within orthogroups may reflect evolutionary processes such as gene family expansion and functional diversification among fungal pathogens.

Phylogenetic analysis showed that all six *U. virens* strains clustered together, indicating a high level of genomic conservation within the species. As expected, the two-outgroup species formed distinct lineages separate from the *U. virens* clade. Among these, *M. oryzae* showed relatively closer phylogenetic proximity to *Ustilaginoidea* compared with *U. maydis*, which is consistent with their taxonomic placement within the phylum Ascomycota, whereas *U. maydis* belongs to Basidiomycota.

In addition, several previously reported *U. virens* effector genes; *UvHrip1*, *Cut1*, *Uv1809*, *UvCBP1*, and *SCRE2*, were screened across all six genomes. The Indian strains UV-GVT and NRRI-FSM-1 contained five and four of these effectors, respectively (with SCRE2 absent in NRRI-FSM-1), whereas only two of effectors, UvHrip1 and Cut1, were detected in iJS62, IPU010, P1, and UV-8b genomes. Secretome analysis across six *U. virens* genomes revealed broadly similar overall secretory architectures, although the proportion of predicted candidate effectors varied among strains. This variability is consistent with previous observations that effector prediction is strongly influenced by the computational tools, prediction thresholds, and annotation pipelines used. Therefore, differences in effector counts should be interpreted with caution. The relatively conserved secretome composition, coupled with variation in effector repertoires, is a common feature of fungal plant pathogens, where conserved secretory systems support diverse effector complements that mediate host–pathogen interactions.

### NRRI-FSM-1 strain harbors extra set of secondary metabolites and CAZymes genes

Through antiSMASH analysis, various classes of secondary metabolites were identified in all six *U. virens* strains, including reference strain UV-8b, and a total of 156 genes from all six strains were identified. Among the individual strains, NRRI-FSM-1 codes for a maximum of 28 and UV-GVT and IPU010 code for 25 each, which is the lowest among all. The reason for this variation in the number of secondary metabolite coding genes in RFS fungal strains may be attributed to different geographical locations and potential variation in their pathogenicity (Xue et al., 2023). Interestingly, NRRI-FSM-1 had the highest number of secondary metabolite genes, whereas the other India strain, UV-GVT had one of the least numbers of these genes. Additionally, NRRI-FSM-1 codes for NAPAA, which is completely absent in UV-GVT. Interestingly, NRRI-FSM-1 codes for five genes belonging to T1PKS, responsible for RFS phytotoxin production, and also higher pathogenicity (Xue et al., 2023).

In this study, further predicted genes coding for carbohydrate-active enzymes (CAZymes), which are the enzymes produced by pathogenic fungi to break down plant cell walls and help in fungal infection and feed on host plants (Zhao et al., 2014). NRRI-FSM-1 codes for highest number of CAZymes, and UV-GVT codes for lowest number of genes from this category. Therefore, this finding gives an indication how NRRI-FSM-1 might differ with other strains, specifically the Indian strain UV-GVT in terms of its arsenal of diverse genes. A comparison of CAZymes across all strains shows NRRI-FSM-1 having approximately 2% more families (18.38%) than any other strain. Additionally, the GT59 family was uniquely present in NRRI-FSM-1, while the GH24 family was absent in this strain. The GT59 family of CAZymes, a glycosyltransferase, catalyzes the synthesis of specific carbohydrate structures. In plant-fungal pathogen interactions, GT59 plays a role in synthesizing crucial carbohydrates having a role in cell wall formation and host-pathogen recognition.

Comparative genome analyses of *Ustilaginoidea virens* strains provide an opportunity to generate testable hypotheses regarding the genetic determinants underlying pathogenicity and regional adaptation. In the present study, the NRRI-FSM-1 genome exhibited a higher number of strain-specific genes, secondary metabolite clusters, and CAZyme families compared with other available genomes. One plausible hypothesis is that specific gene sets, including candidate effectors, toxin biosynthesis genes, and cell wall–degrading enzymes, may contribute to enhanced adaptation of pathogen populations to specific agro-ecological conditions. For instance, the increased representation of secondary metabolite genes and CAZyme families in NRRI-FSM-1 may reflect functional diversification associated with host colonization and toxin production. However, rigorous evaluation of these hypotheses will require population-scale genomic analyses coupled with phenotypic characterization across multiple isolates representing diverse geographic regions and virulence backgrounds. The genome sequence presented here therefore provides an important resource for future comparative pathogenomic studies aimed at identifying virulence determinants and understanding the evolutionary dynamics of *U. virens* populations. In the longer term, integrating genomic surveillance with climate-based risk models and toxicology data will improve forecasting and help prioritize management strategies to reduce yield loss and food-safety risk (Sathiyaseelan et al., 2025).

## Conclusions

The whole-genome sequencing and comparative genomic analysis of the Eastern Indian *Ustilaginoidea virens* strain NRRI-FSM-1 reveal a highly dynamic genome characterized by substantial structural variation and genetic plasticity. The presence of an expanded accessory genome, a high number of presence–absence variations, and strain-specific genes underscores significant genomic diversity and potential regional adaptation within *U. virens* populations. Variation in SNPs, InDels, simple sequence repeats, and transposable elements further highlights ongoing genome evolution. Functional analyses indicate an enriched repertoire of candidate effectors, secondary metabolite gene clusters, and carbohydrate-active enzymes, suggesting an enhanced capacity for host colonization, toxin production, and pathogenicity. Collectively, these findings provide important insights into the evolutionary dynamics of *U. virens* and establish NRRI-FSM-1 as a valuable genomic resource for future studies on pathogen biology, virulence mechanisms, and the development of effective disease management strategies.

## Materials and methods

### Sequencing of *Ustilaginoidea virens* genome

The total genomic DNA from one of the highly virulent strains of *Ustilaginoidea virens* (NRRI-FSM-1) was extracted using modified CTAB method (**Supplementary Data 1**) and its genome was sequenced using Illumina HiSeq 2500 (Xcelris Lab ltd., India) with paired-end reads and a read length of 150 bases. The sequence data has been submitted to NCBI database bearing file No. SRR9618123.sra (https://www.ncbi.nlm.nih.gov/sra/?term=SRR9618123). The whole genome sequencing of this fungus was carried out. NRRI-FSM-1 was originally isolated from an Indian mega indica rice variety Pooja. The data SRA was used and converted to FASTQ using the prefetch and fastq-dump modules of sratoolkit v.2.9.4-2 (https://trace.ncbi.nlm.nih.gov/Traces/sra/sra.cgi?view=software)

### *De novo* whole genome assembly

Quality check of the raw reads was performed using FastQC v.0.11.8 (https://www.bioinformatics.babraham.ac.uk/projects/fastqc/). No read trimming was required because the reads submitted were already trimmed for adapter sequence contamination, and showed a per-base sequence quality average over 30 Phred. The high-quality reads were utilized for the *de novo* whole genome assembly analysis using SOAPdenovo v.2.04 (Luo et al., 2012). Before performing the assembly analysis, a k-mer analysis was carried out on the high-quality reads to determine the best suitable k-mer size. The k-mer analysis was done by KmerGenie v.1.7051 with default parameters (Chikhi and Medvedev, 2014). The SOAPdenovo analysis was then performed with the following parameters: all (which includes pre-graph construction, contig generation, read mapping onto contigs, and scaffold formation), 150 bases maximum read length, 350 bases average library insert size, 105 k-mer size, resolve repeats by reads, and fill gaps in scaffolds. All scaffolds with less than 500 bases were excluded from further analysis. All filtered scaffolds (>=500 bases) were subsequently subjected to the Python tool RagTag (https://github.com/malonge/RagTag/blob/master/ragtag.py) to assign chromosome-wise coordinates using a rice smut fungus reference genome sequence. The reference genome sequence (GCF_000687475.1, Assembly version: ASM68747v2) was retrieved from the NCBI assembly database (https://www.ncbi.nlm.nih.gov/datasets/genome/GCF_000687475.1/). The assembled chromosome-wise sequences were used for further downstream analysis.

### Small variant detection in NRRI-FSM-1

Small variant detection in the rice smut fungal genome enables identification of strain-specific polymorphisms that drive pathogenicity and host adaptation. Thus, small variants, such as single nucleotide polymorphisms (SNPs) and small insertions-deletions (InDels < 50 bp), were identified using a pipeline based on BWA v.0.7.17-r1198-dirty (Li and Durbin, 2009), Samtools v.1.16.1 (Danecek et al., 2021), Picard tools v.2.27.5 (https://broadinstitute.github.io/picard/), and gatk-package-4.1.9.0 (https://github.com/broadinstitute/gatk/releases?page=2) (DePristo et al., 2011). In this analysis, the reference sequence (ASM68747v2) was indexed using the “index” command of BWA, and the processed reads were mapped to the reference sequence with the bwa mem command. The resulting alignment SAM file was converted into a BAM file using the samtools view with “-bS” option. Duplicate reads were marked in the BAM file with the Picard “MarkDuplicates” module, followed by position-based sorting using the samtools sort. Finally, small variants were called using the HaplotypeCaller module of gatk-package. The raw variants were filtered to remove low-quality calls using by the gatk-package with thresholds of base quality >30 (Phred), depth >10, and mapping quality >40. The filtered variants were further annotated using SNPEff v.5.0e (Cingolani et al., 2012).

### Identification of repeats in reported *U. virens* strains

Repeat identification was performed to annotate transposable elements and repetitive DNA, which are important for understanding genome organization and structural variation in the rice smut fungus. Assembled sequences of all six genomes taken from the database [UV-GVT (GenBank No. GCA_002939685.2), iJS62 (GenBank No. GCA_024397435.1), IPU010 (GenBank No. GCA_000965225.2), P1 (GenBank No. GCA_016803955.1), and UV-8b (RefSeq No. GCF_000687475.1)], including the reference genome (Strain UV-8b) and the currently studied genome (Strain NRRI-FSM-1), were passed through RepeatMasker v.4.1.4 in sensitive mode for homology-based masking of the repeat regions in the targeted *U. virens* genome sequences (https://www.repeatmasker.org/RepeatMasker/). The UV2_4G genome reported from India could not be included in the comparative analysis, as its sequence is not publicly available. The *de novo* repeat identification and annotation of repeats were performed using the EDTA package (Ou et al., 2019). The initial repeat library was generated through EDTA and subsequently utilized in the RepeatMasker analysis as a custom repeat library. Prediction of simple sequence repeats (SSRs) from the rice smut fungal genome provides valuable markers for genetic diversity, population structure, and evolutionary studies. Therefore, SSRs were also identified from all six strains using MISA software (Beier et al., 2017) with the following parameters: repeat unit size and minimum number of repeats, 2/10, 3/7, 4/5, 5/4 and 6/4, and 100 nucleotide length difference between two SSRs (Thiel et al., 2003). The assembled sequences were used for the SSRs prediction.

### Gene prediction and functional annotation in reported *U. virens* strains

Gene modules were predicted for each reported *U. virens* genome, separately using the repeat-masked sequences obtained from the repeat analysis. The gene prediction was carried out using Funannotate v.1.8.13 (https://funannotate.readthedocs.io/en/latest/install.html), a dynamic tool that adjusts prediction based on the input parameters passed to the funannotate predict script. At the core of the prediction algorithm is Evidence Modeler, which integrates several different gene prediction inputs to generate consensus gene models. The *ab initio* gene predictors integrated within Funannotate include Augustus, SNAP, GlimmerHMM, CodingQuarry, and GeneMark-ES/ET. An important feature of Funannotate is its ability to incorporate external “evidence” into predicted transcripts. The predicted genes were subsequently analyzed for gene ontology and assigned to their respective functional categories.

Gene ontology and functional categorization analyses were performed to assign standardized biological functions to predicted genes in the rice smut fungal genome. Gene ontology and functional categorization were performed using a set of software tools: BLASTP of DIAMOND v.2.0.4 (Buchfink et al., 2015), InterProScan v.5.62-94.0 (Zdobnov and Apweiler, 2001), and emapper v.2.1.6 of EggNOG (Huerta-Cepas et al., 2019). Carbohydrate-related gene annotation analysis was also conducted using the online tool CAZy (https://www.cazy.org/Home.html) based on the Carbohydrate-Active enZYmes Database (Drula et al., 2022).

### Analysis of *Ustilaginoidea virens* pangenome

Pangenome construction in rice smut fungi is essential for capturing the full spectrum of core and accessory genes, enabling identification of strain-specific effectors, genomic diversity, and evolutionary adaptations that contribute to pathogenicity and host specialization. For this analysis, six *U. virens* assembled genome sequences, including the reference genome and the NRRI-FSM-1 genome, along with their annotation information were used. The pangenome was constructed using ppsPCP software (Qamar et al., 2019). The presence and absence variation (PAV) analysis was also executed with this software using default parameters so as to identify strain-specific genes and genomic regions with similar set of parameters.

### *Ustilaginoidea virens* secretome analysis

Secretory protein analysis is a prerequisite for effector prediction, as candidate effectors must be secreted to interact with host cells. Identifying proteins with signal peptides and secretion pathways helps narrow down the effector repertoire from the whole genome. Secretory proteins were identified using the pipeline developed by Singh et al. (2019). All the proteins of a genome were initially subjected to SignalP 4.1 (Petersen et al., 2011) and TargetP 2.0 (Armenteros et al., 2019) analysis. Results of both TargetP (Loc = S) and SignalP (D-score = Y) analyses were combined to generate a set of consensus sequences obtained from both analyses. The consensus sequences were then passed to the online tool DeepTMHMM v.1.0.24 for scanning transmembrane spanning regions in these proteins (https://dtu.biolib.com/DeepTMHMM). All proteins with 0 TMs or 1 TM, if located in the first 40 amino acids, were considered for further analysis. All GPI-anchor proteins were identified using the big-PI Fungal Predictor (https://mendel.imp.ac.at/gpi/fungi_server.html). After this step, all proteins identified by DeepTMHMM and big-PI were analyzed for their cellular localization using ProtComp v. 9.0, with reference to LocDB (proteins with known localization) and PotLocDB (proteins with strong theoretical evidence of localization) databases (http://www.softberry.com/berry.phtml?topic=protcomppl&group=help&subgroup=proloc) to validate the secreted proteins. All the extracellular, secreted proteins obtained from the ProtComp analysis were considered refined secreted proteins. These refined secreted proteins were further analyzed by using “runWolfPsortSummary fungi” module of WoLF PSORT v.0.2 (Horton et al., 2007) to *in silico* confirm their localization. After obtaining the refined secreted proteins, their functional annotation was performed using InterProScan v.5.62-94.0 (Zdobnov and Apweiler, 2001). All analyses were performed with default parameters. Moreover, the refined secretome datasets were also used to identify candidate effectors using both machine learning–based and similarity search–based methods. In the machine learning approach, EffectorP was employed to detect candidate effectors (Sperschneider and Dodds, 2022). For the similarity search–based approach, the known effector databases DFVF and PHI-base, along with reported effectors of *U. virens*, were merged and utilized (Lu et al., 2012; Urban et al., 2025). The BLASTP of DIAMOND v.2.0.4 was employed to perform the similarity search analysis with 1 maximum target sequence parameter and 10–^5^ e-value cut-off (Buchfink et al., 2015).

### Analysis of *Ustilaginoidea virens* secondary metabolite

Secondary metabolite prediction was performed using online tool antiSMASH v.7.0.1 with complete genomic sequences of each *U. virens* strain (Blin et al., 2023). This tool provides rapid genome-wide identification, annotation, and analysis of secondary metabolite biosynthesis gene clusters in bacterial and fungal genomes. It integrates and cross-links with a large number of *in silico* secondary metabolite analysis tools that have been previously utilized for secondary metabolite analysis.

### Comparative analysis of *Ustilaginoidea virens* strains

Orthologous identification is required in comparative genome analysis to establish gene correspondences across strains, enabling accurate assessment of conserved regions, and evolutionary relationships. Orthologous identification in all six *U. virens* strains, along with two outgroup species, *Magnaporthe oryzae* and *Ustilago maydis*, was done using OrthoFinder v.2.5.4 with default parameters (Emms and Kelly, 2019). The total protein sequences of each strain were used as input for the orthologous analysis. This analysis involved five major steps: protein alignment, protein clustering, construction of a tree library, distance matrix generation of orthogroups, and final tree construction. These steps were performed by DIAMOND (Buchfink et al., 2015), the MCL algorithm (van Dongen, 2000), ETE (Huerta-Cepas et al., 2016), DendroBLAST (Kelly and Maini, 2013), and FastME (Lefort et al., 2015), respectively, in the background to obtain the final output.

### Visualization of the output data

The results obtained from various analyses of all six Ustilaginoidea genomes were visualized using Circos v0.69-8 (Krzywinski et al., 2009) and SyMap v5.5.6 (Soderlund et al., 2006). For the Circos plots, the density of SNPs and InDels per 100 kb in the NRRI-FSM-1 genomic regions was calculated separately for each chromosome. In SyMap-based synteny visualization, the seven chromosomes of the reference genome UV-8b were aligned in pairwise mode with the corresponding chromosomal contigs of NRRI-FSM-1 that had already been assigned to specific chromosomes. The UpSet plot was generated using the web-based tool Hiplot (https://hiplot.cn/basic/upset-plot) to represent common and unique orthologs identified across *U. maydis*, *M. oryzae*, NRRI-FSM-1, UV-GVT, IPU010, P1, iJS62, and UV-8b. In contrast, the bubble plot, heatmap, and stacked bar plot were constructed using custom Python scripts. Secondary metabolite biosynthetic gene clusters present in six *U. virens* strains (NRRI-FSM-1, UV-GVT, iJS62, IPU010, P1, and UV-8b) were represented by the bubble plot. Similarly, the CAZyme family distribution in these six *U. virens* strains was shown in the heatmap, whereas the SSR distribution was displayed using the stacked bar plot.

## Data Availability

The datasets presented in this study can be found in online repositories. The names of the repository/repositories and accession number(s) can be found below: https://www.ncbi.nlm.nih.gov/, SRR9618123.
