# Supplementary material for: Comparative genomics of Eastern-Indian Ustilaginoidea virens strain NRRI-FSM-1 at whole-genome level unravels genome evolution and genetic plasticity
Source: Front Fungal Biol. 2026 May 21;7:1828327. doi: 10.3389/ffunb.2026.1828327 (PMC13233505; doi:10.3389/ffunb.2026.1828327)
Supplement: Supplementary file 1 [file DataSheet1.docx]

Supplementary Figure 1. Results of secretome analysis and their comparison among six rice smut fungi. The x-axis shows the number of proteins obtained at each step of the analysis, and the y-axis lists the software tools used.

Supplementary Table 1. Distribution of CAZyme families among the smut strains

| CAZyme Family | NRRI-FSM-1 | UV-GVT | iJS62 | IPU010 | P1 | UV-8b | Total |
| --- | --- | --- | --- | --- | --- | --- | --- |
| AA1 | 8 | 7 | 6 | 7 | 6 | 7 | 41 |
| AA11 | 3 | 3 | 3 | 3 | 3 | 3 | 18 |
| AA12 | 1 | 1 | 1 | 1 | 1 | 1 | 6 |
| AA2 | 4 | 4 | 4 | 4 | 4 | 4 | 24 |
| AA3 | 6 | 5 | 6 | 6 | 6 | 6 | 35 |
| AA4 | 3 | 3 | 3 | 3 | 3 | 3 | 18 |
| AA5 | 4 | 2 | 2 | 2 | 2 | 2 | 14 |
| AA6 | 1 | 1 | 1 | 1 | 1 | 1 | 6 |
| AA7 | 5 | 4 | 5 | 4 | 5 | 5 | 28 |
| AA8 | 6 | 2 | 3 | 3 | 3 | 3 | 20 |
| AA9 | 1 | 1 | 1 | 1 | 1 | 1 | 6 |
| CBM21 | 1 | 1 | 1 | 1 | 1 | 1 | 6 |
| CE1 | 2 | 2 | 2 | 2 | 2 | 2 | 12 |
| CE16 | 1 | 1 | 1 | 1 | 1 | 1 | 6 |
| CE3 | 1 | 1 | 1 | 1 | 1 | 1 | 6 |
| CE4 | 3 | 3 | 3 | 2 | 3 | 3 | 17 |
| CE5 | 3 | 4 | 4 | 4 | 4 | 4 | 23 |
| CE8 | 2 | 2 | 2 | 2 | 2 | 2 | 12 |
| CE9 | 1 | 1 | 1 | 1 | 1 | 1 | 6 |
| GH1 | 1 | 1 | 1 | 1 | 1 | 1 | 6 |
| GH10 | 1 | 1 | 1 | 1 | 1 | 1 | 6 |
| GH11 | 1 | 1 | 1 | 1 | 1 | 1 | 6 |
| GH12 | 1 | 1 | 1 | 1 | 1 | 1 | 6 |
| GH125 | 2 | 2 | 2 | 2 | 2 | 2 | 12 |
| GH128 | 2 | 2 | 2 | 2 | 2 | 2 | 12 |
| GH13 | 6 | 6 | 6 | 5 | 6 | 6 | 35 |
| GH132 | 2 | 2 | 2 | 2 | 2 | 2 | 12 |
| GH135 | 1 | 1 | 1 | 1 | 1 | 1 | 6 |
| GH15 | 2 | 2 | 2 | 2 | 2 | 2 | 12 |
| GH105 | 0 | 1 | 1 | 1 | 1 | 1 | 5 |
| GH152 | 1 | 1 | 1 | 1 | 1 | 1 | 6 |
| GH154 | 1 | 1 | 1 | 1 | 1 | 1 | 6 |
| GH16 | 15 | 15 | 15 | 15 | 15 | 15 | 90 |
| GH17 | 2 | 2 | 2 | 2 | 2 | 2 | 12 |
| GH179 | 2 | 2 | 2 | 2 | 2 | 2 | 12 |
| GH18 | 11 | 11 | 11 | 11 | 11 | 11 | 66 |
| GH2 | 3 | 3 | 3 | 3 | 4 | 3 | 19 |
| GH20 | 1 | 1 | 1 | 1 | 1 | 1 | 6 |
| GH24 | 0 | 1 | 1 | 1 | 1 | 1 | 5 |
| GH28 | 4 | 4 | 4 | 4 | 3 | 4 | 23 |
| GH3 | 8 | 6 | 6 | 6 | 6 | 6 | 38 |
| GH31 | 6 | 4 | 5 | 5 | 5 | 5 | 30 |
| GH32 | 2 | 2 | 2 | 2 | 2 | 2 | 12 |
| GH33 | 1 | 1 | 1 | 1 | 1 | 1 | 6 |
| GH35 | 1 | 1 | 1 | 1 | 1 | 1 | 6 |
| GH36 | 2 | 2 | 2 | 2 | 2 | 2 | 12 |
| GH37 | 2 | 1 | 1 | 1 | 1 | 1 | 7 |
| GH38 | 2 | 1 | 1 | 1 | 1 | 1 | 7 |
| GH43 | 1 | 1 | 1 | 1 | 1 | 1 | 6 |
| GH47 | 11 | 9 | 9 | 9 | 9 | 9 | 56 |
| GH5 | 6 | 5 | 4 | 4 | 4 | 4 | 27 |
| GH55 | 7 | 5 | 5 | 5 | 5 | 5 | 32 |
| GH62 | 1 | 1 | 1 | 1 | 1 | 1 | 6 |
| GH63 | 1 | 1 | 1 | 1 | 2 | 1 | 7 |
| GH64 | 2 | 2 | 2 | 2 | 2 | 2 | 12 |
| GH65 | 1 | 1 | 1 | 1 | 1 | 1 | 6 |
| GH72 | 7 | 6 | 6 | 6 | 6 | 6 | 37 |
| GH75 | 1 | 1 | 1 | 1 | 1 | 1 | 6 |
| GH76 | 13 | 9 | 9 | 9 | 9 | 9 | 58 |
| GH81 | 1 | 1 | 1 | 1 | 1 | 1 | 6 |
| GH89 | 1 | 1 | 1 | 1 | 1 | 1 | 6 |
| GH92 | 2 | 2 | 2 | 2 | 2 | 2 | 12 |
| GT1 | 6 | 5 | 5 | 5 | 5 | 5 | 31 |
| GT15 | 4 | 4 | 4 | 4 | 4 | 4 | 24 |
| GT17 | 1 | 1 | 1 | 1 | 1 | 1 | 6 |
| GT2 | 18 | 14 | 15 | 14 | 15 | 15 | 91 |
| GT20 | 4 | 4 | 4 | 4 | 4 | 4 | 24 |
| GT21 | 1 | 1 | 1 | 1 | 1 | 1 | 6 |
| GT22 | 4 | 4 | 4 | 3 | 4 | 4 | 23 |
| GT24 | 1 | 1 | 1 | 1 | 1 | 1 | 6 |
| GT3 | 1 | 1 | 1 | 1 | 1 | 1 | 6 |
| GT31 | 1 | 1 | 0 | 0 | 1 | 1 | 4 |
| GT32 | 2 | 2 | 2 | 2 | 2 | 2 | 12 |
| GT33 | 1 | 1 | 1 | 1 | 1 | 1 | 6 |
| GT34 | 2 | 2 | 2 | 2 | 2 | 2 | 12 |
| GT35 | 1 | 1 | 1 | 1 | 1 | 1 | 6 |
| GT39 | 3 | 3 | 3 | 3 | 3 | 3 | 18 |
| GT4 | 4 | 4 | 4 | 4 | 4 | 4 | 24 |
| GT48 | 2 | 1 | 1 | 0 | 1 | 1 | 6 |
| GT50 | 1 | 1 | 1 | 1 | 1 | 1 | 6 |
| GT57 | 2 | 2 | 2 | 2 | 2 | 2 | 12 |
| GT58 | 1 | 1 | 1 | 1 | 1 | 1 | 6 |
| GT59 | 1 | 0 | 0 | 0 | 0 | 0 | 1 |
| GT62 | 3 | 3 | 3 | 3 | 3 | 3 | 18 |
| GT64 | 1 | 1 | 1 | 2 | 1 | 1 | 7 |
| GT66 | 1 | 1 | 1 | 1 | 1 | 1 | 6 |
| GT69 | 1 | 0 | 1 | 1 | 1 | 1 | 5 |
| GT76 | 1 | 1 | 1 | 1 | 1 | 1 | 6 |
| GT77 | 1 | 1 | 1 | 0 | 0 | 1 | 4 |
| GT8 | 4 | 2 | 4 | 4 | 3 | 4 | 21 |
| GT90 | 6 | 4 | 4 | 4 | 4 | 4 | 26 |
| Total | 273 | 239 | 244 | 239 | 244 | 246 | 1485 |

Supplementary Table 2. Candidate effectors identified using the machine learning method

| NRRI-FSM-1 | UV-GVT | iJS62 | IPU010 | P1 | UV-8b |
| --- | --- | --- | --- | --- | --- |
| NRRI_000804-T1 (A) | GVT_007058-T1 (A) | IJS62_007290-T1 (A) | IPU010_000083-T1 (A) | P1_007291-T1 (A) | UV8B_006781-T1 (A) |
| NRRI_000951-T1 (A) | GVT_000041-T1 (A) | IJS62_007613-T1 (A) | IPU010_000402-T1 (A) | P1_007610-T1 (A) | UV8B_007284-T1 (A) |
| NRRI_002804-T1 (A) | GVT_000862-T1 (A) | IJS62_000021-T1 (A) | IPU010_002836-T1 (A) | P1_000211-T1 (A) | UV8B_007610-T1 (A) |
| NRRI_003486-T1 (A) | GVT_000867-T1 (A) | IJS62_000226-T1 (A) | IPU010_002939-T1 (A) | P1_000225-T1 (A) | UV8B_000022-T1 (A) |
| NRRI_004598-T1 (A) | GVT_000984-T1 (A) | IJS62_000400-T1 (A) | IPU010_003088-T1 (A) | P1_000397-T1 (A) | UV8B_000224-T1 (A) |
| NRRI_004968-T1 (A) | GVT_001020-T1 (A) | IJS62_001062-T1 (A) | IPU010_000088-T1 (A) | P1_001062-T1 (A) | UV8B_000398-T1 (A) |
| NRRI_005192-T1 (A) | GVT_001086-T1 (A) | IJS62_001338-T1 (A) | IPU010_000937-T1 (A) | P1_001336-T1 (A) | UV8B_001061-T1 (A) |
| NRRI_006035-T1 (A) | GVT_001692-T1 (A) | IJS62_001650-T1 (A) | IPU010_000943-T1 (A) | P1_002868-T1 (A) | UV8B_001335-T1 (A) |
| NRRI_006279-T1 (A) | GVT_002329-T1 (A) | IJS62_002249-T1 (A) | IPU010_001014-T1 (A) | P1_003077-T1 (A) | UV8B_002235-T1 (A) |
| NRRI_007579-T1 (A) | GVT_002948-T1 (A) | IJS62_003077-T1 (A) | IPU010_001132-T1 (A) | P1_003095-T1 (A) | UV8B_003079-T1 (A) |
| NRRI_007735-T1 (A) | GVT_003561-T1 (A) | IJS62_003096-T1 (A) | IPU010_001236-T1 (A) | P1_003111-T1 (A) | UV8B_003081-T1 (A) |
| NRRI_008614-T1 (A) | GVT_003773-T1 (A) | IJS62_003154-T1 (A) | IPU010_002323-T1 (A) | P1_003152-T1 (A) | UV8B_003135-T1 (A) |
| NRRI_009194-T1 (A) | GVT_004300-T1 (A) | IJS62_003230-T1 (A) | IPU010_004238-T1 (A) | P1_003234-T1 (A) | UV8B_003214-T1 (A) |
| NRRI_009272-T1 (A) | GVT_004857-T1 (A) | IJS62_003235-T1 (A) | IPU010_004400-T1 (A) | P1_003239-T1 (A) | UV8B_003219-T1 (A) |
| NRRI_009304-T1 (A) | GVT_004983-T1 (A) | IJS62_003416-T1 (A) | IPU010_004536-T1 (A) | P1_003422-T1 (A) | UV8B_003403-T1 (A) |
| NRRI_000105-T1 (A) | GVT_005052-T1 (A) | IJS62_004453-T1 (A) | IPU010_005099-T1 (A) | P1_004461-T1 (A) | UV8B_004442-T1 (A) |
| NRRI_000203-T1 (A) | GVT_005118-T1 (A) | IJS62_004832-T1 (A) | IPU010_005534-T1 (A) | P1_004843-T1 (A) | UV8B_004820-T1 (A) |
| NRRI_000369-T1 (A) | GVT_005736-T1 (A) | IJS62_005029-T1 (A) | IPU010_005806-T1 (A) | P1_004849-T1 (A) | UV8B_005018-T1 (A) |
| NRRI_000615-T1 (A) | GVT_006152-T1 (A) | IJS62_005080-T1 (A) | IPU010_005886-T1 (A) | P1_005042-T1 (A) | UV8B_005069-T1 (A) |
| NRRI_000710-T1 (A) | GVT_006209-T1 (A) | IJS62_005247-T1 (A) | IPU010_006234-T1 (A) | P1_005094-T1 (A) | UV8B_005235-T1 (A) |
| NRRI_000749-T1 (A) | GVT_006444-T1 (A) | IJS62_005606-T1 (A) | IPU010_006932-T1 (A) | P1_005258-T1 (A) | UV8B_005593-T1 (A) |
| NRRI_000819-T1 (A) | GVT_006686-T1 (A) | IJS62_005614-T1 (A) | IPU010_007098-T1 (A) | P1_005703-T1 (A) | UV8B_005600-T1 (A) |
| NRRI_001330-T1 (A) | GVT_007328-T1 (A) | IJS62_005698-T1 (A) | IPU010_007332-T1 (A) | P1_006135-T1 (A) | UV8B_005682-T1 (A) |
| NRRI_001362-T1 (A) | GVT_007361-T1 (A) | IJS62_006132-T1 (A) | IPU010_007530-T1 (A) | P1_006569-T1 (A) | UV8B_006114-T1 (A) |
| NRRI_001462-T1 (A) | GVT_007416-T1 (A) | IJS62_006567-T1 (A) | IPU010_001119-T1 (A) | P1_007133-T1 (A) | UV8B_001045-T1 (A) |
| NRRI_002403-T1 (A) | GVT_000611-T1 (A) | IJS62_007130-T1 (A) | IPU010_002054-T1 (A) | P1_007168-T1 (A) | UV8B_001538-T1 (A) |
| NRRI_002472-T1 (A) | GVT_001046-T1 (A) | IJS62_007167-T1 (A) | IPU010_002119-T1 (A) | P1_001046-T1 (A) | UV8B_001599-T1 (A) |
| NRRI_002821-T1 (A) | GVT_001364-T1 (A) | IJS62_007189-T1 (A) | IPU010_004222-T1 (A) | P1_001541-T1 (A) | UV8B_001622-T1 (A) |
| NRRI_003266-T1 (A) | GVT_001435-T1 (A) | IJS62_007710-T1 (A) | IPU010_004591-T1 (A) | P1_001607-T1 (A) | UV8B_002415-T1 (A) |
| NRRI_003275-T1 (A) | GVT_003272-T1 (A) | IJS62_000305-T1 (A) | IPU010_004885-T1 (A) | P1_001634-T1 (A) | UV8B_003115-T1 (A) |
| NRRI_003438-T1 (A) | GVT_003616-T1 (A) | IJS62_001046-T1 (A) | IPU010_006486-T1 (A) | P1_002254-T1 (A) | UV8B_003325-T1 (A) |
| NRRI_003509-T1 (A) | GVT_003789-T1 (A) | IJS62_001543-T1 (A) | IPU010_007492-T1 (A) | P1_002434-T1 (A) | UV8B_005003-T1 (A) |
| NRRI_003568-T1 (A) | GVT_003965-T1 (A) | IJS62_001604-T1 (A) | IPU010_002709-T1 (C) | P1_003132-T1 (A) | UV8B_001060-T1 (C) |
| NRRI_003679-T1 (A) | GVT_005081-T1 (A) | IJS62_001632-T1 (A) | IPU010_000747-T1 (C) | P1_003345-T1 (A) | UV8B_003901-T1 (C) |
| NRRI_003770-T1 (A) | GVT_005486-T1 (A) | IJS62_002430-T1 (A) | IPU010_001133-T1 (C) | P1_005028-T1 (A) | UV8B_004492-T1 (C) |
| NRRI_004071-T1 (A) | GVT_005646-T1 (A) | IJS62_003134-T1 (A) | IPU010_001256-T1 (C) | P1_006796-T1 (A) | UV8B_004652-T1 (C) |
| NRRI_005279-T1 (A) | GVT_006259-T1 (A) | IJS62_003338-T1 (A) | IPU010_002093-T1 (C) | P1_003923-T1 (C) | UV8B_005019-T1 (C) |
| NRRI_005609-T1 (A) | GVT_000184-T1 (C) | IJS62_005015-T1 (A) | IPU010_003947-T1 (C) | P1_004494-T1 (C) | UV8B_006311-T1 (C) |
| NRRI_006017-T1 (A) | GVT_000272-T1 (C) | IJS62_006797-T1 (A) | IPU010_004237-T1 (C) | P1_004513-T1 (C) | UV8B_006526-T1 (C) |
| NRRI_006662-T1 (A) | GVT_001412-T1 (C) | IJS62_003089-T1 (C) | IPU010_005533-T1 (C) | P1_004675-T1 (C) | UV8B_000679-T1 (B) |
| NRRI_006992-T1 (A) | GVT_001554-T1 (C) | IJS62_003910-T1 (C) | IPU010_005938-T1 (C) | P1_005043-T1 (C) | UV8B_000783-T1 (B) |
| NRRI_007384-T1 (A) | GVT_002317-T1 (C) | IJS62_004505-T1 (C) | IPU010_007370-T1 (C) | P1_006333-T1 (C) | UV8B_003575-T1 (B) |
| NRRI_007389-T1 (A) | GVT_003342-T1 (C) | IJS62_004663-T1 (C) | IPU010_007403-T1 (C) | P1_006550-T1 (C) | UV8B_003628-T1 (B) |
| NRRI_007429-T1 (A) | GVT_005702-T1 (C) | IJS62_005030-T1 (C) | IPU010_000588-T1 (B) | P1_006979-T1 (C) | UV8B_003741-T1 (B) |
| NRRI_007644-T1 (A) | GVT_007327-T1 (C) | IJS62_006330-T1 (C) | IPU010_001174-T1 (B) | P1_007152-T1 (C) | UV8B_004243-T1 (B) |
| NRRI_007841-T1 (A) | GVT_007655-T1 (C) | IJS62_006548-T1 (C) | IPU010_001268-T1 (B) | P1_007266-T1 (C) | UV8B_004656-T1 (B) |
| NRRI_008084-T1 (A) | GVT_000257-T1 (B) | IJS62_006980-T1 (C) | IPU010_001409-T1 (B) | P1_000679-T1 (B) | UV8B_005135-T1 (B) |
| NRRI_008370-T1 (A) | GVT_000570-T1 (B) | IJS62_007148-T1 (C) | IPU010_001514-T1 (B) | P1_000782-T1 (B) | UV8B_005157-T1 (B) |
| NRRI_008842-T1 (A) | GVT_000684-T1 (B) | IJS62_007255-T1 (C) | IPU010_001705-T1 (B) | P1_003594-T1 (B) | UV8B_005159-T1 (B) |
| NRRI_008953-T1 (A) | GVT_000757-T1 (B) | IJS62_000681-T1 (B) | IPU010_001750-T1 (B) | P1_003647-T1 (B) | UV8B_005434-T1 (B) |
| NRRI_009319-T1 (A) | GVT_001213-T1 (B) | IJS62_000783-T1 (B) | IPU010_003184-T1 (B) | P1_003763-T1 (B) | UV8B_005553-T1 (B) |
| NRRI_000234-T1 (A) | GVT_001280-T1 (B) | IJS62_003586-T1 (B) | IPU010_003980-T1 (B) | P1_004266-T1 (B) | UV8B_005630-T1 (B) |
| NRRI_008042-T1 (C) | GVT_001915-T1 (B) | IJS62_003639-T1 (B) | IPU010_004022-T1 (B) | P1_004679-T1 (B) | UV8B_006514-T1 (B) |
| NRRI_000818-T1 (C) | GVT_002651-T1 (B) | IJS62_003752-T1 (B) | IPU010_004917-T1 (B) | P1_005158-T1 (B) | UV8B_003054-T1 (B) |
| NRRI_001033-T1 (C) | GVT_002811-T1 (B) | IJS62_004252-T1 (B) | IPU010_005209-T1 (B) | P1_005180-T1 (B) |  |
| NRRI_001389-T1 (C) | GVT_002831-T1 (B) | IJS62_004667-T1 (B) | IPU010_005222-T1 (B) | P1_005182-T1 (B) |  |
| NRRI_001977-T1 (C) | GVT_003635-T1 (B) | IJS62_005147-T1 (B) | IPU010_005224-T1 (B) | P1_005575-T1 (B) |  |
| NRRI_002202-T1 (C) | GVT_003960-T1 (B) | IJS62_005156-T1 (B) | IPU010_005333-T1 (B) | P1_005650-T1 (B) |  |
| NRRI_002267-T1 (C) | GVT_004009-T1 (B) | IJS62_005169-T1 (B) | IPU010_005342-T1 (B) | P1_006538-T1 (B) |  |
| NRRI_002451-T1 (C) | GVT_005046-T1 (B) | IJS62_005171-T1 (B) | IPU010_005985-T1 (B) | P1_006681-T1 (B) |  |
| NRRI_002864-T1 (C) | GVT_005566-T1 (B) | IJS62_005522-T1 (B) | IPU010_006016-T1 (B) | P1_006765-T1 (B) |  |
| NRRI_002974-T1 (C) | GVT_005720-T1 (B) | IJS62_005567-T1 (B) | IPU010_006044-T1 (B) | P1_007013-T1 (B) |  |
| NRRI_003066-T1 (C) | GVT_005722-T1 (B) | IJS62_006536-T1 (B) | IPU010_006730-T1 (B) | P1_007036-T1 (B) |  |
| NRRI_003510-T1 (C) | GVT_006791-T1 (B) | IJS62_006682-T1 (B) | IPU010_006984-T1 (B) | P1_007056-T1 (B) |  |
| NRRI_004069-T2 (C) | GVT_007388-T1 (B) | IJS62_006765-T1 (B) | IPU010_007193-T1 (B) | P1_007124-T1 (B) |  |
| NRRI_004758-T1 (C) | GVT_007665-T1 (B) | IJS62_007013-T1 (B) | IPU010_003481-T1 (B) | P1_007214-T1 (B) |  |
| NRRI_005971-T1 (C) |  | IJS62_007036-T1 (B) |  | P1_007242-T1 (B) |  |
| NRRI_007142-T1 (C) |  | IJS62_007056-T1 (B) |  | P1_007265-T1 (B) |  |
| NRRI_007375-T1 (C) |  | IJS62_007120-T1 (B) |  | P1_003070-T1 (B) |  |
| NRRI_008632-T1 (C) |  | IJS62_007129-T1 (B) |  |  |  |
| NRRI_009009-T1 (C) |  | IJS62_007213-T1 (B) |  |  |  |
| NRRI_005950-T1 (B) |  | IJS62_007241-T1 (B) |  |  |  |
| NRRI_000089-T1 (B) |  | IJS62_007265-T1 (B) |  |  |  |
| NRRI_000172-T1 (B) |  | IJS62_003070-T1 (B) |  |  |  |
| NRRI_000202-T1 (B) |  |  |  |  |  |
| NRRI_000722-T1 (B) |  |  |  |  |  |
| NRRI_000763-T1 (B) |  |  |  |  |  |
| NRRI_000920-T1 (B) |  |  |  |  |  |
| NRRI_001196-T1 (B) |  |  |  |  |  |
| NRRI_001242-T1 (B) |  |  |  |  |  |
| NRRI_001316-T1 (B) |  |  |  |  |  |
| NRRI_001328-T1 (B) |  |  |  |  |  |
| NRRI_001389-T2 (B) |  |  |  |  |  |
| NRRI_002058-T1 (B) |  |  |  |  |  |
| NRRI_002437-T1 (B) |  |  |  |  |  |
| NRRI_002866-T1 (B) |  |  |  |  |  |
| NRRI_003073-T1 (B) |  |  |  |  |  |
| NRRI_003677-T1 (B) |  |  |  |  |  |
| NRRI_004001-T1 (B) |  |  |  |  |  |
| NRRI_005953-T1 (B) |  |  |  |  |  |
| NRRI_006373-T1 (B) |  |  |  |  |  |
| NRRI_006576-T1 (B) |  |  |  |  |  |
| NRRI_006589-T1 (B) |  |  |  |  |  |
| NRRI_006650-T1 (B) |  |  |  |  |  |
| NRRI_007141-T1 (B) |  |  |  |  |  |
| NRRI_007512-T1 (B) |  |  |  |  |  |
| NRRI_008321-T1 (B) |  |  |  |  |  |
| (A)- Apoplastic effector, (C)- Cytoplasmic effector, (B)- Both aploplastic/cytoplasmic effector | | | | | |

Supplementary Table 3. List of candidate effectors identified in NRRI-FSM-1 through similarity search

| Protein | Known effector | Description of matched known effector |
| --- | --- | --- |
| NRRI_000804-T1 | Hsp150p | E7Q5D7#PHI:5043#Hsp150p#4932#Saccharomyces_cerevisiae#increased_virulence_(hypervirulence) |
| NRRI_001616-T1 | Crh1 | A0A384J6C4#PHI:11358#Crh1_(Bcin01g06010)#40559#Botrytis_cinerea#effector_(plant_avirulence_determinant) |
| NRRI_002804-T1 | VLP4 | J4KLP6#PHI:7192#VLP4#176275#Beauveria_bassiana#reduced_virulence |
| NRRI_002985-T1 | BcEG | A0A384JRK6 #PHI:10433#BcEG_(Bcin09g00200)#40559#Botrytis_cinerea#unaffected_pathogenicity_reduced_virulence |
| NRRI_005192-T1 | GLX | A0A0E0SJJ3#PHI:5393#GLX#5518#Fusarium_graminearum#reduced_virulence |
| NRRI_005549-T1 | CfmC | Q4WNE1#PHI:4017#CfmC#746128#Aspergillus_fumigatus#unaffected_pathogenicity |
| NRRI_006279-T1 | FGSG_01588 | I1RD96#PHI:3984#FGSG_01588_(Sc_gpi7)#5518#Fusarium_graminearum#unaffected_pathogenicity |
| NRRI_006861-T1 | Sap2 | P0CS83#PHI:124142#Sap2#5476#Candida_albicans#increased_virulence_(hypervirulence) |
| NRRI_007691-T1 | Eng1 | J4W7P1#PHI:124316#Eng1_(BBA_04753)#176275#Beauveria_bassiana#reduced_virulence_increased_virulence_(hypervirulence) |
| NRRI_007735-T1 | MrSVP | E9ESA9#PHI:8804#MrSVP_(EFZ01626)#568076#Metarhizium_robertsii#reduced_virulence |
| NRRI_008014-T1 | BcEG | A0A384JRK6#PHI:10433#BcEG_(Bcin09g00200)#40559#Botrytis_cinerea#unaffected_pathogenicity_reduced_virulence |
| NRRI_008277-T1 | AdsA | A0A391DK74#PHI:12218#AdsA#1280#Staphylococcus_aureus#reduced_virulence |
| NRRI_008335-T1 | BcCFEM1 | A6SR05#PHI:7594#BcCFEM1#40559#Botrytis_cinerea#reduced_virulence_unaffected_pathogenicity |
| NRRI_9357-T1 | FGSG_01588 | I1RD96#PHI:3984#FGSG_01588_(Sc_gpi7)#5518#Fusarium_graminearum#unaffected_pathogenicity |
| NRRI_000085-T1 | Aph1 | J9VHR6#PHI:3236#Aph1#5207#Cryptococcus_neoformans#reduced_virulence |
| NRRI_000087-T1 | Gh92 | D5AK25#PHI:10565#Gh92#1307#Streptococcus_suis#reduced_virulence |
| NRRI_000105-T1 | LysM1 | A0A0A2ILW0#PHI:7664#LysM1#27334#Penicillium_expansum#effector_(plant_avirulence_determinant) |
| NRRI_000170-T1 | LIP1 | Q5XTQ4#PHI:541#LIP1#40559#Botrytis_cinerea#unaffected_pathogenicity |
| NRRI_000267-T1 | FgPR-IL-2 | I1RG88#PHI:7649#FgPR-IL-2#5518#Fusarium_graminearum#unaffected_pathogenicity |
| NRRI_000734-T1 | FET3-2 | E3Q7G #PHI:2921_PHI:9075#FET3-2_Fet3-2#31870#Colletotrichum_graminicola#reduced_virulence |
| NRRI_000746-T1 | Bab2 | Q2YIV2#PHI:9126#Bab2_0277#235#Brucella_abortus#reduced_virulence |
| NRRI_001665-T1 | FAED2 | A0A194VKY8#PHI:10225#FAED2_(VM1G_00901)#105487#Valsa_mali#unaffected_pathogenicity |
| NRRI_002121-T1 | BcBGL4 | A0A384JDM0#PHI:10368#BcBGL4_(BCIN_03g08710)#40559#Botrytis_cinerea#reduced_virulence |
| NRRI_002151-T1 | Tyr | G4MP53#PHI:123880#Tyr(MGG_14598)#318829#Magnaporthe_oryzae#reduced_virulence |
| NRRI_002451-T1 | FoSOD5 | X0LE71#PHI:11164#FoSOD5_(FOTG_08628)#5507#Fusarium_oxysporum#reduced_virulence |
| NRRI_002821-T1 | FGSG_08238 | I1RVG3#PHI:124250#FGSG_08238#5518#Fusarium_graminearum#unaffected_pathogenicity |
| NRRI_002976-T1 | FGSG_11472 | I1S3S6#PHI:5900#FGSG_11472#5518#Fusarium_graminearum#unaffected_pathogenicity |
| NRRI_003175-T1 | Cgyps1 | Q6FJR #PHI:7925#Cgyps1#5478#Candida_glabrata#reduced_virulence_unaffected_pathogenicity |
| NRRI_003438-T1 | Blys8 | J4KL74#PHI:7381#Blys8#176275#Beauveria_bassiana#unaffected_pathogenicity |
| NRRI_003509-T1 | SRE1 | R0K2C5#PHI:123376#SRE1_(SETTUDRAFT_163271)#93612#Exserohilum_turcicum#effector_(plant_avirulence_determinant) |
| NRRI_003770-T1 | MoXYL1B | G4NA54#PHI:2214_PHI:123401#Endo-1_4-beta-xylanase_I_GH10_family_MoXYL1B_(MGG_08424)#318829#Magnaporthe_oryzae#reduced_virulence_unaffected_pathogenicity_loss_of_pathogenicity |
| NRRI_003779-T1 | SpnA | A0A455ZLU4 #PHI:9098#SpnA#1314#Streptococcus_pyogenes#reduced_virulence |
| NRRI_003806-T1 | FGSG_03402 | I1RHY5#PHI:6125_PHI:5716#FGSG_03402_ScOrtholog_PHO11#5518#Fusarium_graminearum#unaffected_pathogenicity |
| NRRI_003832-T1 | Tyr | G4MP53 #PHI:123880#Tyr(MGG_14598)#318829#Magnaporthe_oryzae#reduced_virulence |
| NRRI_003966-T1 | Cgfl | E3QKL1#PHI:12176#Cgfl_(GLRG_06543)#31870#Colletotrichum_graminicola#effector_(plant_avirulence_determinant) |
| NRRI_004033-T2 | Man1 | G4ND25 #PHI:2488#Man1#318829#Magnaporthe_oryzae#unaffected_pathogenicity |
| NRRI_004122-T1 | Aorsin | J4KLI2#PHI:124352#Aorsin_(BBA_08791)#176275#Beauveria_bassiana#increased_virulence_(hypervirulence) |
| NRRI_004253-T1 | Chi2 | Q4U4T0#PHI:2388#Chi2#5530#Metarhizium_anisopliae#increased_virulence_(hypervirulence)_reduced_virulence |
| NRRI_004511-T3 | GLX | X0J8P9 #PHI:5393#GLX#5507#Fusarium_oxysporum#reduced_virulence |
| NRRI_004782-T1 | FvSCP | W7M3S4 #PHI:7144#FvSCP1#117187#Fusarium_verticillioides#reduced_virulence |
| NRRI_005101-T1 | CHT42 | O59928 #PHI:144#CHT42#29875#Trichoderma_virens#reduced_virulence |
| NRRI_005411-T1 | Cpcat1 | O60038 #PHI:1034#Cpcat1#5111#Claviceps_purpurea#unaffected_pathogenicity |
| NRRI_005435-T1 | BbepnL-1 | J4UIJ0 #PHI:9546#BbepnL-1_(BBA_07766)#176275#Beauveria_bassiana#reduced_virulence |
| NRRI_005710-T1 | MoAPX2 | G4MZ98 #PHI:5186#MoAPX2#318829#Magnaporthe_oryzae#reduced_virulence |
| NRRI_005997-T1 | UM01888.1 | Q4PDC5_USTMA UM01888.1 Ustilago maydis PF00069 Pkinase Smut |
| NRRI_006017-T1 | CDA | L2FHG9 #PHI:3972#CDA#474922#Colletotrichum_gloeosporioides#unaffected_pathogenicity |
| NRRI_006150-T1 | BcBGL3 | A0A384JWC5 #PHI:10367#BcBGL3_(BCIN_10g05590)#40559#Botrytis_cinerea#reduced_virulence |
| NRRI_006158-T1 | PGX1 | Q96VZ3 #PHI:181#PGX1#5507#Fusarium_oxysporum#unaffected_pathogenicity |
| NRRI_006565-T1 | Cut1 | A0A8E5HQL5#PHI:124205#Cut1_(Uv8b_03824)#1159556#Ustilaginoidea_virens#increased_virulence_(hypervirulence) |
| NRRI_006663-T1 | Eng1 | C0NFK7 #PHI:6265#Eng1#5037#Histoplasma_capsulatum#reduced_virulence |
| NRRI_007140-T1 | CLU5c | E3QFA6 #PHI:10751#CLU5c_(GLRG_04688)#31870#Colletotrichum_graminicola#unaffected_pathogenicity |
| NRRI_007141-T1 | Uv1809 | A0A8E5HR16 #PHI:124328#Uv1809#1159556#Ustilaginoidea_virens#reduced_virulence |
| NRRI_007142-T1 | MC69 | G5EI17 #PHI:3122#MC69#318829#Magnaporthe_oryzae#effector_(plant_avirulence_determinant) |
| NRRI_007389-T1 | SIX2 | Q709E0 #PHI:5285#SIX2#5507#Fusarium_oxysporum#effector_(plant_avirulence_determinant) |
| NRRI_007470-T1 | CUT1 | CUTI_ERYGR CUT1 Erysiphe graminis PF00964 Elicitin Powdery Mildew |
| NRRI_007841-T1 | Sm1 | Q0R411 #PHI:6417#Sm1#29875#Trichoderma_virens#reduced_virulence |
| NRRI_008051-T1 | FGSG_12142 | I1S5M1 #PHI:5903#FGSG_12142#5518#Fusarium_graminearum#unaffected_pathogenicity |
| NRRI_008323-T1 | PGX1 | Q00359 #PHI:115#PGX1#5017#Bipolaris_zeicola#unaffected_pathogenicity |
| NRRI_008357-T1 | Gtb1 | L7JCL9 #PHI:10459#Gtb1#318829#Magnaporthe_oryzae#reduced_virulence |
| NRRI_008953-T1 | UvHrip1 | A0A1B5KZM5 #PHI:10357_PHI:10826#UvHrip1_(UVI_02019870)#1159556#Ustilaginoidea_virens#effector_(plant_avirulence_determinant) |
| NRRI_009023-T1 | UvCBP1 | UvCBP1 tr\|A0A1B5KX72\|A0A1B5KX72_USTVR \|UvCBP1 chitinase OS=Ustilaginoidea virens OX=1159556 GN=UVI_02019410 PE=3 SV=1 |
| NRRI_009077-T1 | MoAa91 | G4N560 #PHI:10358#MoAa91_(MGG_06069)#318829#Magnaporthe_oryzae#reduced_virulence |
| NRRI_009167-T1 | Penlp2 | A0A0A2JGQ7 #PHI:9299#Penlp2_(PEX2_071150)#27334#Penicillium_expansum#unaffected_pathogenicity |
| NRRI_000923-T1 | Cgps5 | Q6FVH8 #PHI:7929#Cgyps5#5478#Candida_glabrata#reduced_virulence_unaffected_pathogenicity |
| NRRI_001155-T1 | Gel1 | G4MV25 #PHI:6711#Gel1#318829#Magnaporthe_oryzae#unaffected_pathogenicity_loss_of_pathogenicity |
| NRRI_001197-T1 | GAS1 | Q2KN79 #PHI:522_PHI:2808#GAS1_Gas1#5507#Fusarium_oxysporum#reduced_virulence |
| NRRI_001433-T1 | Plb1 | Q8TG07 #PHI:3600#Plb1#5478#Candida_glabrata#unaffected_pathogenicity |
| NRRI_002316-T1 | RI_SAPP2 | G8B6Y8 #PHI:9401#RI_SAPP2_(CPAR2_102580)#5480#Candida_parapsilosis#unaffected_pathogenicity_reduced_virulence |
| NRRI_004002-T2 | GAS1 | Q2KN79 #PHI:522_PHI:2808#GAS1_Gas1#5507#Fusarium_oxysporum#reduced_virulence |
| NRRI_004828-T1 | MoFLP1 | G5EHM3 #PHI:4231#MoFLP1#318829#Magnaporthe_oryzae#reduced_virulence |
| NRRI_007920-T1 | UM01886.1 | Q4PDC7_USTMA UM01886.1 Ustilago maydis PF03142 Chitin_synth_2 Smut |
| NRRI_008248-T1 | Gas1 | X0I5S4 #PHI:6530#Gas1#5507#Fusarium_oxysporum#reduced_virulence |
| NRRI_008770-T1 | Gel5 | G4NAB0 #PHI:6715#Gel5#318829#Magnaporthe_oryzae#unaffected_pathogenicity |
| Purple-colored proteins represent common effectors identified by both the machine learning and similarity search methods. Red-colored effectors indicate their presence in *U. virens*. | | |

Fungal DNA isolation protocol

Fungal mycelia were scraped and ground into a fine powder using liquid nitrogen. Approximately 200 mg of finely ground mycelial powder was transferred into a 2 mL microcentrifuge tube, followed by the addition of 600 µL CTAB extraction buffer. The mixture was thoroughly homogenized by vortexing. The tubes were then incubated in a water bath at 65 °C for 30 min, followed by cooling at room temperature for 10 min. The samples were subsequently centrifuged at 13,000 rpm for 10 min, and the supernatant was transferred to a fresh 2 mL microcentrifuge tube, and an equal volume (v/v) of chloroform:isoamyl alcohol (24:1) was added. The mixture was gently mixed by inversion and centrifuged at 13,000 rpm for 15 min. The aqueous phase was carefully transferred to a fresh 1.5 mL microcentrifuge tube, followed by the addition of an equal volume of chilled isopropanol (v/v). The tubes were gently mixed and centrifuged at 13,000 rpm for 15 min to precipitate the DNA.

Composition of CTAB buffer (for 100 mL buffer)

| Component | Working concentration | Stock Amount |
| --- | --- | --- |
| CTAB (Cetyltrimethylammonium bromide) | 2.2 % (w/v) | 2 g |
| Tris-HCl (pH 8.0) | 100 mM | 10 mL of 1 M |
| EDTA (pH 8.0) | 20 mM | 4 mL of 0.5 M |
| NaCl | 1.4 M | 8.2 g |
| PVP-40 (Polyvinylpyrrolidone) | 1% (optional but recommended) | 1 g |
| Sterile distilled water | — | to 100 mL |

* Add freshly before use β-mercaptoethanol (0.3%)
